# Oxa-Ferrier Rearrangement Reaction Mediated by TEMPO Cation and NaClO_2_: Application to the Total Synthesis of Passifetilactones B and C

**DOI:** 10.1021/acs.joc.5c00354

**Published:** 2025-04-28

**Authors:** Jocelyn Bautista-Nava, Luis F. Porras-Santos, Leticia Quintero, José Alvano Pérez-Bautista, Pedro López-Mendoza, Fernando Sartillo-Piscil

**Affiliations:** Centro de Investigación de la Facultad de Ciencias Químicas, Benemérita Universidad Autónoma de Puebla (BUAP), 14 Sur Esq. San Claudio, Col. San Manuel, 72570, Puebla, México

## Abstract

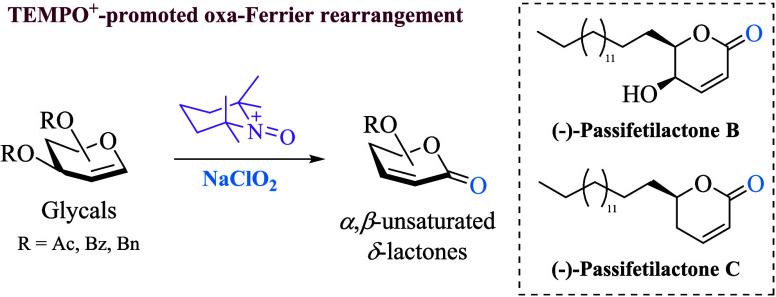

The TEMPO^+^ (2,2,6,6-tetramethylpiperidine-*N*-oxyl cation) is a versatile chemical species commonly known as an oxidizing reagent. Nevertheless, its capability to act as a Lewis acid has been recently revealed. Here, we report a TEMPO^+^-promoted oxa-Ferrier rearrangement of glycals to chiral α,β-unsaturated δ-lactones using sodium chlorite (NaClO_2_) as a cheap and environmentally friendly oxidizing reagent. Since the vinylic oxocarbenium intermediate is trapped by chlorite ion to form a carbonyl group, we name this reaction as the “Oxa-Ferrier rearrangement”. Accordingly, this reaction is suitable for various *O*-acetylated, *O*-benzoylated, and *O*-benzylated glycals, providing the corresponding α,β-unsaturated δ-lactones in moderate to good yield. Additionally, the synthetic utility of this methodology was applied to the synthesis and confirmation of the absolute configuration of passifetilactones B and C.

## Introduction

**Scheme 1:**
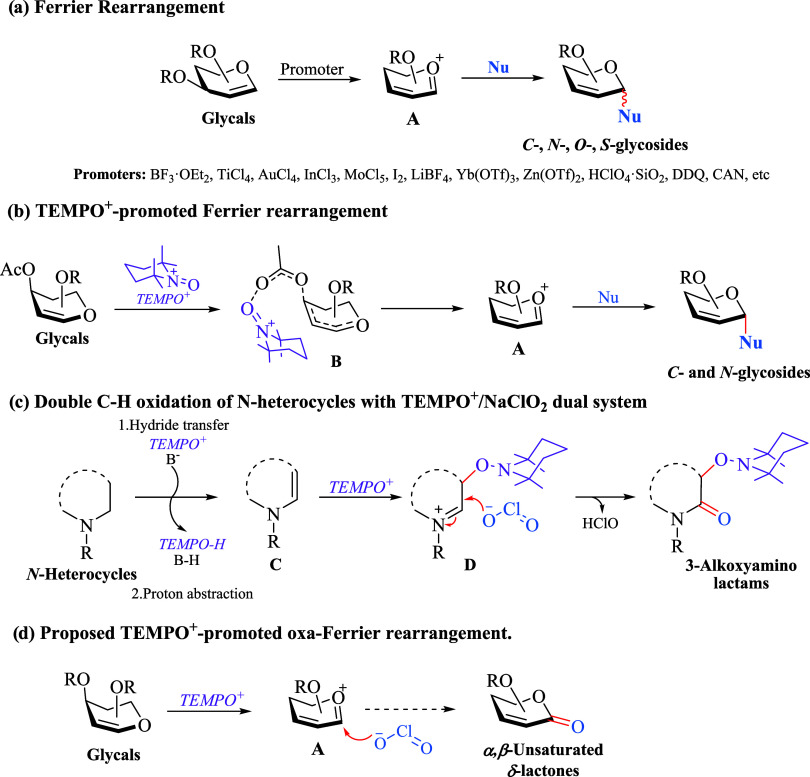
Conception of the Proposed Oxa-Ferrier Rearrangement Reaction

The Ferrier rearrangement is a powerful chemical reaction that enables the stereoselective formation of 2,3-unsaturated glycosides from simple acetylated glycals.^1^ Hence, it has been widely applied in the total synthesis of natural products and biologically active compounds.^2^ The reaction mechanism involves the formation of a vinylogous oxocarbenium ion intermediate **A**, which is attacked by *C*-, *N*-, *O*-, and *S*-nucleophiles to generate the corresponding *C*-, *N*-, *O*-, and *S*-glycosides (Scheme 1a).

**Scheme 2:**
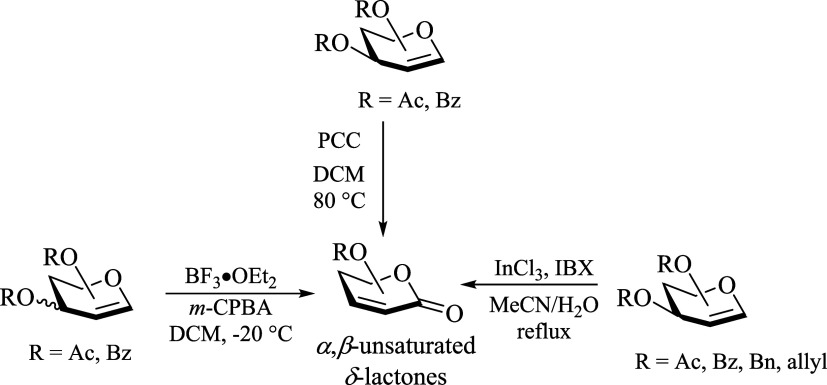
Synthesis of Chiral α, β-Unsaturated *δ*-Lactones from Glycals

Several promoters^3^ such as Brønsted acids (HClO_4_·SiO_2_ and HBF_4_·SiO_2_),^4^ Lewis acids (BF_3_·OEt_2_, TiCl_4_, AuCl_3_, InCl_3_, LiBF_4_, Yb(OTf)_3_, Zn(OTf)_2_),^5^ and oxidants (DDQ and CAN)^6^ have been reported (Scheme 1a); nevertheless, we recently discovered that TEMPO^+^ catalyzes the Ferrier rearrangement reaction to *C*- and *N*-glycosides.^7^ Based on experimental and theoretical studies, it was proved that the glycal-TEMPO^+^ mesomeric structure **B** promotes the formation of **A**, and hence, a type of Lewis acid activation was proposed (Scheme 1b).^7,8^ Despite this unprecedented behavior of TEMPO^+^ is quite contrasting with the C–H functionalization of *N*-heterocycles to 3-alkoxyamino lactams via enamine **C** (Scheme 1c),^9^ we anticipated that the nucleophilic attack of chlorite ion to **D** could occur similar to **A**, and thus to form the *α,β*-unsaturated δ-lactones from glycals (Scheme 1d). Accordingly, this nucleophilic addition of chlorite anion to vinylogous oxocarbenium ion intermediate **A**, can be referred to as an oxa-Ferrier rearrangement reaction. Evidently, this chemical reaction could be an interesting alternative to those previously reported, which have found application to the synthesis of bioactive compounds containing the chiral α,β-unsaturated δ-lactone moiety.^10^ Indeed, similar oxidations of glycals to α,β-unsaturated δ-lactones have been documented,^11^ albeit with some issues to deal with, such as the inherent toxicity associated with the use of pyridinium chlorochromate (PCC),^12^ the incompatibility of some protecting groups in the presence of strong, air and moisture sensitive Lewis acids like BF_3_·OEt_2_,^13^ or even the use of expensive promoters like InCl_3_^14^ (Scheme 2). Consequently, since the TEMPO^+^ salt (and analogues thereof) acts as a nonmetallic/air and moisture-stable Lewis acid, and the NaClO_2_ is a very cheap and nontoxic oxidizing reagent, the title chemical reaction represents an economical and environmentally friendly alternative for the synthesis of optically active α,β-unsaturated δ-lactones from simple glycals.

## Results and Discussion

To test the proposed oxa-Ferrier rearrangement reaction, 3,4,6-tri-*O*-acetyl-d-glucal (**1**) was selected as the model substrate. Thus, when **1** was subjected to react with 3.0 and 2.0 equiv of TEMPO^+^BF_4_^–^ and NaClO_2_, respectively, in anhydrous acetonitrile (MeCN) at 70 °C, the expected α,β-unsaturated δ-lactone **2** was obtained in 34% yield after stirring for 4 h (Table 1, entry 1). Pleased by this result, we proceeded to optimize the reaction conditions. First, the stoichiometric ratio of the reagents was evaluated. A considerable improvement in the yield was obtained by increasing the equivalents of TEMPO^+^BF_4_^–^ up to 3.5 (entry 2), giving **2** in 50% yield after 2 h.

**Table 1:**
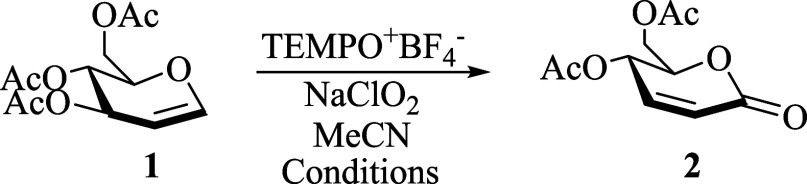
Optimization of the Reaction Conditions

entry	TEMPO^+^BF_4_^–^ (equiv)	NaClO_2_ (equiv)	temperature^a^ (°C)	time	yield^b^ (%)
1^c^	3.0	2.0	70	4.0 h	34
2	3.5	2.0	70	2.0 h	50
3	4.0	2.0	70	2.0 h	53
4	3.5	2.5	70	2.0 h	48
5	3.5	2.0	90	20 min	60
**6**	**3.5**	**1.5**	**90**	**20 min**	**68**
7^d^	3.5	1.5	90	20 min	NR
8^e^	3.5	1.5	90	20 min	NR
9		1.5	90	20 min	NR
10	3.5		90	20 min	0
11^f^	3.5	1.5	90	20 min	60

a All reactions were performed in an oil bath.

b Isolated yield.

c Reaction conditions: **1** (0.092 mmol), **TEMPO**^+^**BF**_**4**_^**–**^ (0.276 mmol), **NaClO**_**2**_ (0.184 mmol) in anhydrous MeCN [0.5 M] at 70 °C under N_2_ atmosphere.

d 1,2-dichloroethane was used as a solvent.

e Toluene was used as a solvent.

f The reaction was performed at 1.25 mmol scale.

While by adding more TEMPO^+^BF_4_^–^ the chemical yield of **2** was not improved significantly (entry 3); it remained almost the same by increasing equivalents of NaClO_2_ up to 2.5 (entry 4). Interestingly, when the reaction temperature was raised to 90 °C, the product was obtained in 60% yield in only 20 min (entry 5). Performing the reaction at this temperature, 1.5 equiv of NaClO_2_ was required to obtain the best chemical yield (entry 6). Additionally, when the reaction was performed in other solvents such as 1,2-dichloroethane and toluene, the reaction did not take place, and the starting material was recovered (entries 7 and 8). Some control experiments were carried out to demonstrate the role of both TEMPO^+^BF_4_^–^ and NaClO_2_. Accordingly, in the absence of TEMPO^+^BF_4_^–^, the starting material remained unchanged (entry 9), meanwhile, without NaClO_2_, only decomposition of the starting material was observed (entry 10). Finally, the reaction was performed at a higher scale (1.25 mmol) under the best reaction conditions, and compound **2** was obtained in 60% yield (entry 11).

Having established the optimal conditions, the scope for this TEMPO^+^-mediated oxa-Ferrier rearrangement was evaluated with various *O*-substituted glycals (Table 2). Accordingly, 3,4,6-tri-*O*-acetyl-D-galactal (**3**) was transformed into the α,β-unsaturated δ-lactone **4** in a moderate 46% yield (Table 2, entry 1). On the other hand, lactones **6** and **8** were obtained in good yields from the C-6 deoxygenated glycals 3,4-di-*O*-acetyl-D-rhamnal (**5**) and 3,4-di-*O*-acetyl-D-fucal (**7**), respectively (entries 2 and 3). Similar results were obtained from 3,4-di-*O*-acetyl-D-xylal (**9**) to compound **10** (entry 4). The same α,β-unsaturated lactone **10** was also obtained from 3,4-di-*O*-acetyl-D-arabinal (**11**) in a comparable good yield (entry 5). Also, C6 functionalized glycals, such as tosylated **12** and iodinated **14**, were tested, giving the corresponding α,β-unsaturated lactones **13** and **15**, respectively, in fair yields (entries 6 and 7). Next, the effect of the C-3 leaving group (benzoyl and benzyl) was also studied. Hence, 3,4,6-tri-*O*-benzoyl-d-glucal (**16**) and 3,4,6-tri-*O*-benzoyl-D-galactal (**18**) gave their corresponding products **17** and **19**, respectively, in modest chemical yields (entries 8 and 9). Similarly, the benzyl group (**20**) gave the same modest chemical yield of **21** (entry 10), proving that both benzoyl and benzyl are poor leaving groups under the current reaction conditions. Finally, TBSO-protected glucal **22** was tested, however, the formation of lactone **23** was not observed. This is probably due to the greater steric repulsion imposed by the TEMPO cation, which largely affects the required SiO---O=N^+^ interaction for promoting the formation of vinylogous oxocarbenium ion intermediate.

**Table 2:**
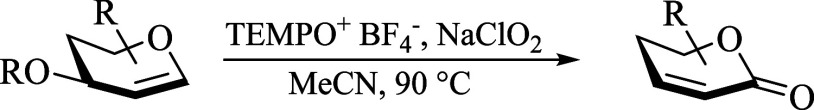

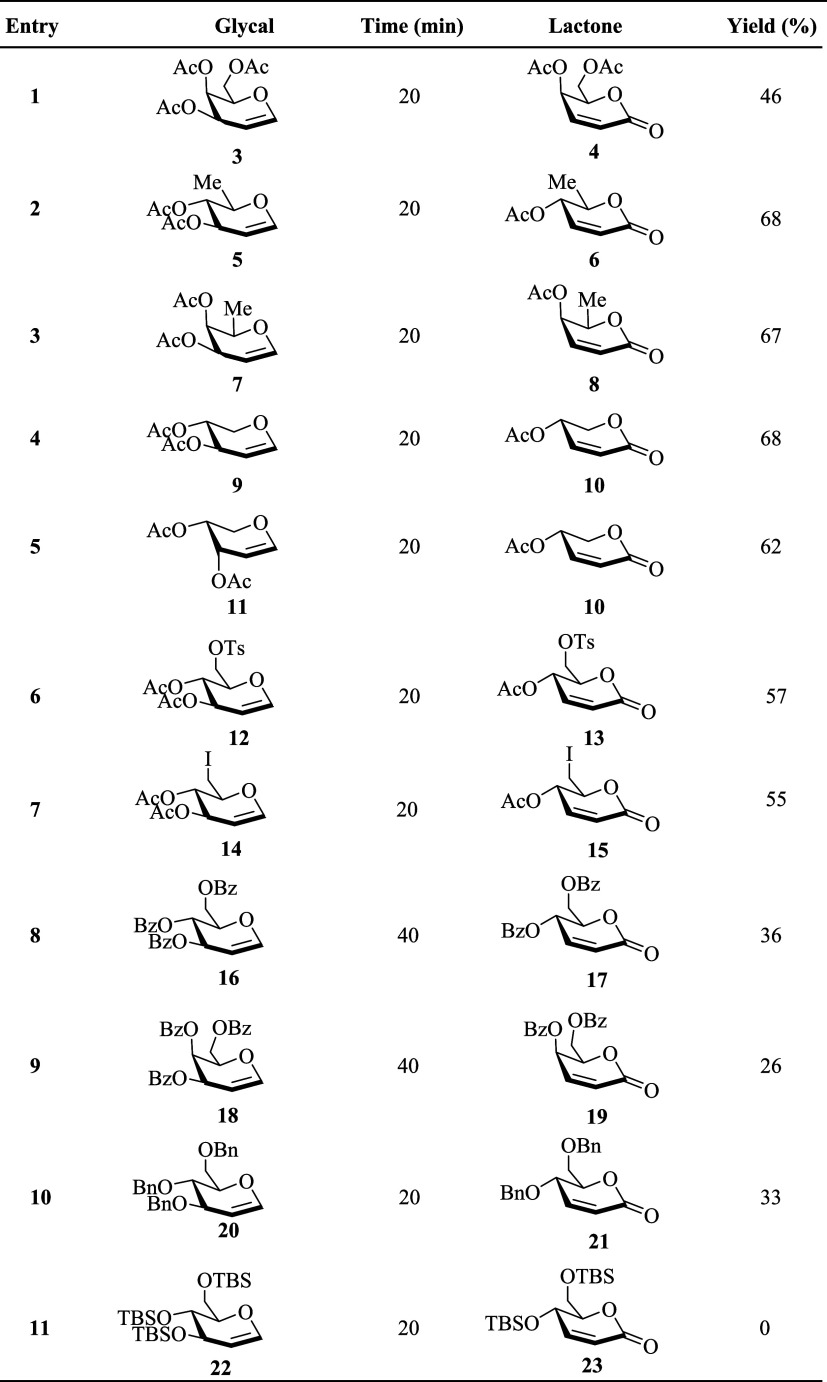
Scope of the TEMPO^+^-Mediated Oxa-Ferrier Rearrangement^a^

a **Reaction conditions:** Glycal (1.0 equiv),TEMPO^+^BF4^–^ (3.5 equiv), and NaClO_2_ (1.5 equiv) in MeCN [0.5 M] at 90 °C under N_2_ atmosphere.

**Scheme 3:**
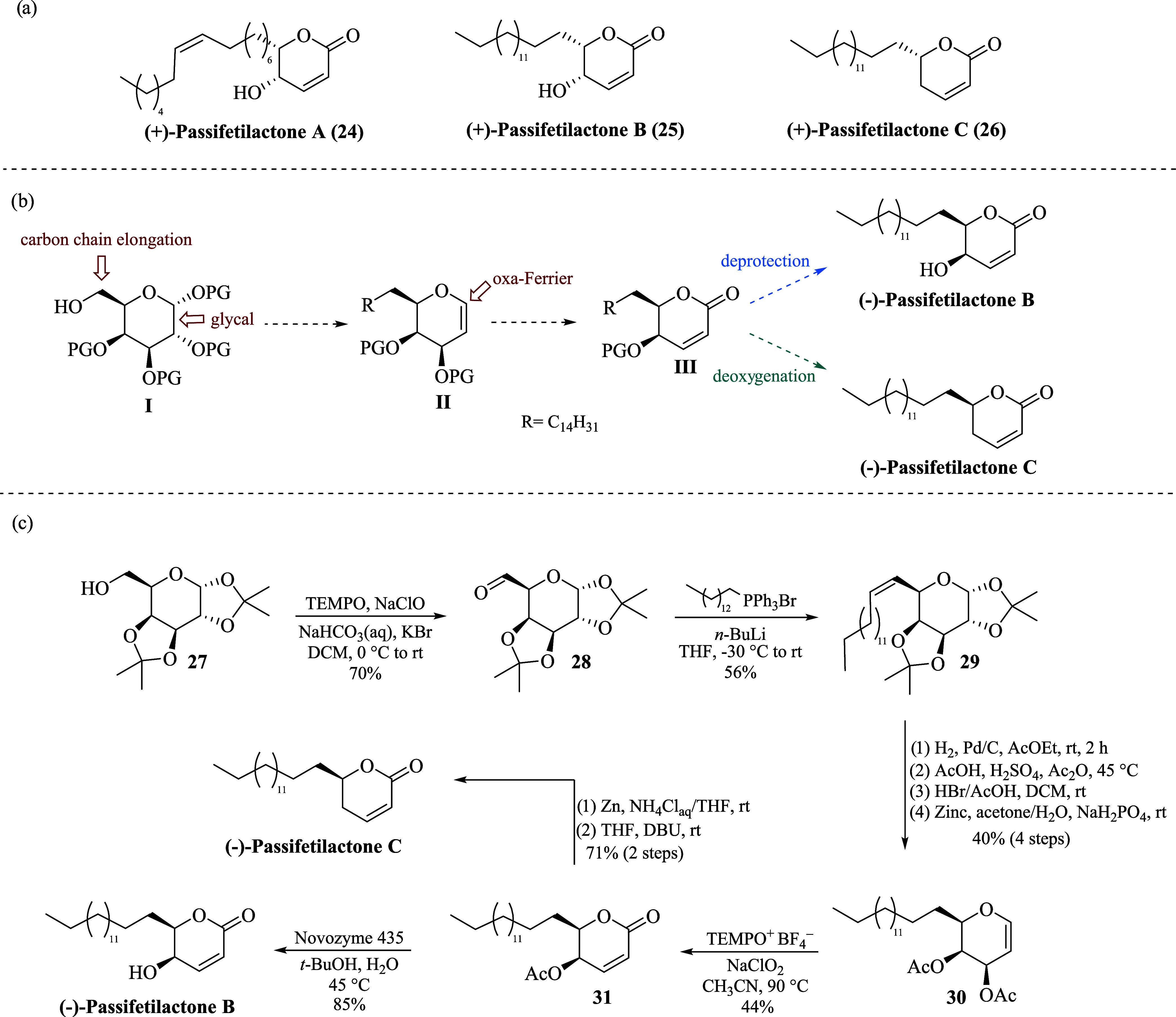
(a) Passifetilactones A–C. (b) Synthetic Strategy to Passifetilactones B and C. (c) Synthesis of Passifetilactones B and C

Based on these results, we decided to apply this novel synthetic protocol to the total synthesis of two recently isolated bioactive natural products (**24**-**26**). Accordingly, (+)-passifetilactone B (**25**) and (+)-passifetilactone C (**26**), two members of a family of fatty acid lactones isolated from the fruit and flowers of *Passiflora fetida* by Schevenels and co-workers, were selected as targets (Scheme 3a).^15^ These natural products displayed moderate to good cytotoxic activity against HeLa (human cervical cancer), A549 (human lung carcinoma), PC-3 (human prostatic cancer), KKU-055, and KKU-213A (cholangiocarcinoma) cell lines.

The synthetic plan to passifetilactones B and C includes initial carbon chain elongation at C-6 of galactopyranose protected derivative **I**, followed by glycal formation to obtain **II**, which will be subjected to the oxa-Ferrier rearrangement to thus obtain the key α,β-unsaturated lactone **III**. Eventually, both (−)-passifetilactone B and (−)-passifetilactone C, the putative enantiomers of the proposed structures, will be obtained by simple deprotection and deoxygenation processes, respectively (Scheme 3b).

Following the synthetic plan, we started oxidizing 1,2:3,4-Di-*O*-isopropylidene-α-D-galactopyranose **27** to aldehyde **28** with NaOCl and catalytic amounts of 2,2,6,6-tetramethylpiperidinyloxy (TEMPO).^16^ Then, Wittig olefination reaction of **28** with *n*-tetradecyl triphenylphosphonium bromide, in the presence of *n*-BuLi, gave **29** in 56% yield.^17^ Hydrogenation of the double bond under catalytic conditions (Pd/C), followed by hydrolysis/acetylation *in situ* afforded the respective tetraacetylated galactopyranose derivative (not shown), which was subsequently treated with HBr/AcOH and reduced with Zn in the presence of NaH_2_PO_4_ to obtain the galactal derivative **30** in 40% yield after four steps and only one column purification.^18^ With glycal **30** in hand, the key oxa-Ferrier rearrangement reaction was successfully applied, and the expected α,β-unsaturated δ-lactone **31** was obtained in 44% yield. Finally, the total synthesis of (−)-passifetilactone B was obtained by applying enzymatic deacetylation with Novozyme 435.^19^ On the other hand, (−)-passifetilactone C was obtained after Zn-promoted reduction, followed by isomerization of the double bond with 1,8-Diazabicyclo[5.4.0]undec-7-ene (DBU).^20^ Spectroscopic NMR data for (−)-passifetilactone B and (−)-passifetilactone C agree with those reported by Schevenels and co-workers (see Tables S1–S4 in Supporting Information). However, although the sign of the specific rotation for both compounds are consistent with those expected for the enantiomers {[α]_*D*_^25^ = −66.0 (c 0.333, MeOH) for synthetic (−)-passifetilactone B, and [α]_*D*_^25^ = −24.0 (c 0.353, MeOH) for synthetic (−)-passifetilactone C} of the natural (+)-passifetilactone B {[α]_*D*_^21^ = +8.0 (c 0.1, MeOH)} and (+)-passifetilactone C {[α]_*D*_^21^ = +2.0 (c 0.1, MeOH)}, there is a significant difference between the value of the synthetic material with those natural products.

## Conclusions

In summary, we have expanded the synthetic application of TEMPO^+^ cation as a Lewis acid, and therefore, a novel oxa-Ferrier rearrangement reaction has been developed using NaClO_2_ as nucleophilic oxidant. Because the TEMPO^+^ salt is a nonmetallic/air and moisture-stable Lewis acid, and the NaClO_2_ is a very cheap and nontoxic oxidizing reagent, this novel, simple and environmentally friendly method to obtain optically active α,β-unsaturated δ-lactones from glycals offers an attractive alternative to the existing methods. In addition, this methodology was applied to the first total synthesis of (−)-passifetilactone B and (−)-passifetilactone C, from which we were able to confirm the absolute configuration of the isolated natural products.

## Experimental Part

### General Information

All reactions were carried out under a nitrogen atmosphere with dry solvents under anhydrous conditions, unless otherwise noted. Commercially available reagents were purchased from Sigma-Aldrich and used without further purification unless otherwise noted. Acetonitrile, tetrahydrofuran (THF), dichloromethane (DCM), and ethyl acetate were used as reactive grade, dried under standard techniques, and freshly distilled prior to use. NaClO_2_ was purchased from MEYER as technical grade (80%). Starting materials were prepared following reported procedures. TEMPO^+^BF_4_^–^ was prepared following the procedure reported by Su.^21^ Column chromatography (CC) was performed using silica gel 230–400 mesh as the stationary phase and a mixture of solvents as the mobile phase, as indicated. Reactions were monitored by thin layer chromatography on 0.25 mm Merk silica gel 60-F254 plates using UV light (multiband UV-256/366 nm), anisaldehyde, ammonium molybdate, and potassium permanganate stain as visualizing agents. Melting points were carried out on a Fisher-Scientific 12–144 melting point apparatus and are not corrected. NMR spectra were recorded on Bruker-500 (500 MHz) using as reference: TMS (0.0 ppm for ^1^H) and residual solvent peak of CDCl_3_ (δ = 7.26 ppm for ^1^H NMR and δ = 77.16 ppm for ^13^C); chemical shifts (δ) are reported in parts per million (ppm) and the coupling constants (*J*) in Hertz (Hz). The following abbreviations (or combinations thereof) were used to explain the multiplicities: s = singlet, d = doublet, t = triplet, q = quartet, m = multiplet, br = broadened. High-resolution mass spectra-electron impact mode (HRMS-EI). High-resolution mass spectra-fast atom bombardment mode (HRMS-FAB).

### General procedure for oxa-Ferrier rearrangement mediated by TEMPO^+^

In a flame-dried sealed tube, glycal (1.0 equiv), TEMPO^+^BF_4_^–^ (3.5 equiv), and sodium chlorite (80 wt %, 1.5 equiv) were dissolved in anhydrous CH_3_CN (molarity of the limiting reagent: [0.5 M]) under a nitrogen atmosphere. The reaction mixture was stirred and heated in an oil bath at 90 °C for a predetermined time (*t*). The values of *t* for each glycal are listed in Table 2. Upon completion of the reaction, the solvent was removed under vacuum, and the residue was purified by flash column chromatography on silica gel using a hexane/EtOAc as eluent system.

#### ((2*R*,3*S*)-3-Acetoxy-6-oxo-3,6-dihydro-2*H*-pyran-2-yl)methyl acetate (**2**)

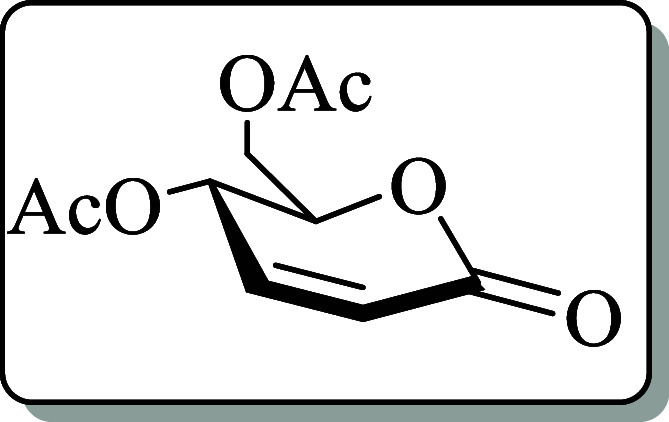



Compound **2** was obtained following the general procedure using **1** (25.0 mg, 0.092 mmol, 1.0 equiv) as starting material, NaClO_2_ (80% w/w, 15.5 mg, 0.136 mmol, 1.5 equiv) and TEMPO^+^ BF_4_^–^ (78.0 mg, 0.322 mmol, 3.5 equiv) in anhydrous acetonitrile (0.18 mL, [0.5 M]). The crude was purified by flash column chromatography on silica gel (hexane/ethyl acetate,7:3) to obtain compound **2** (14.3 mg, 68% yield) as a colorless oil. R_f_ = 0.23 (silica gel, hexane/ethyl acetate, 7:3). ^**1**^**H NMR** (500 MHz, CDCl_3_) δ 6.77 (dd, *J* = 10.0, 3.1 Hz, 1H), 6.09 (dd, *J* = 9.9, 1.8 Hz, 1H), 5.51 (ddd, *J* = 7.9, 3.2, 1.7 Hz, 1H), 4.63 (dt, *J* = 8.2, 4.2 Hz, 1H), 4.31 (dd, *J* = 12.4, 4.6 Hz, 1H), 4.23 (dd, *J* = 12.4, 3.6 Hz, 1H), 2.11 (s, 3H), 2.06 (s, 3H). ^**13**^**C{**^**1**^**H} NMR** (125 MHz, CDCl_3_) δ 170.5, 169.8, 161.2, 143.3, 122.4, 77.4, 63.5, 62.1, 20.8, 20.7. Spectroscopic data agree with those previously reported.^22^

#### ((2*R*,3*R*)-3-Acetoxy-6-oxo-3,6-dihydro-2*H*-pyran-2-yl) methyl acetate (**4**)

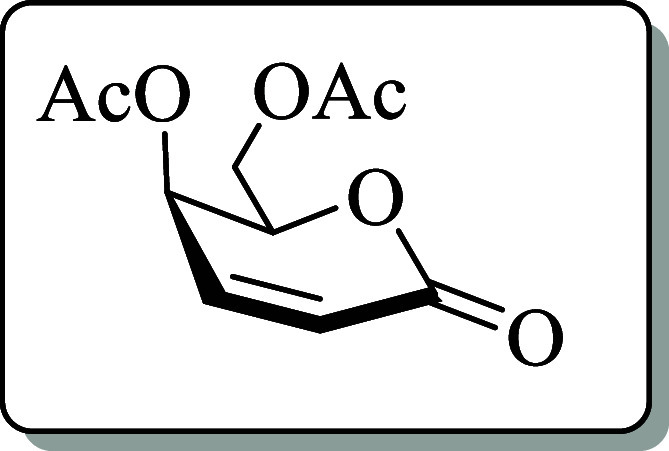



Compound **4** was obtained following the general procedure using **3** (25.0 mg, 0.092 mmol, 1.0 equiv) as starting material, NaClO_2_ (80% w/w, 15.5 mg, 0.136 mmol, 1.5 equiv), TEMPO^+^ BF_4_^–^ (78.0 mg, 0.322 mmol, 3.5 equiv) in anhydrous acetonitrile (0.18 mL, [0.5 M]). The crude was purified by flash column chromatography on silica gel (hexane/ethyl acetate,7:3) to obtain compound **4** (9.6 mg, 46% yield) as a colorless oil. R_f_ = 0.15 (silica gel, hexane/ethyl acetate, 7:3). ^**1**^**H NMR** (500 MHz, CDCl_3_) δ 7.01 (dd, *J* = 9.7, 5.8 Hz, 1H), 6.25 (d, *J* = 9.8 Hz, 1H), 5.30 (dd, *J* = 5.9, 2.8 Hz, 1H), 4.77 (td, *J* = 6.5, 2.8 Hz, 1H), 4.37–4.35 (m, 2H), 2.11 (s, 3H), 2.10 (s, 3H). ^**13**^**C{**^**1**^**H} NMR** (125 MHz, CDCl_33_) δ 170.6, 170.0, 161.7, 140.0, 125.3, 76.0, 61.7, 61.5, 20.8, 20.6. Spectroscopic data agree with those previously reported.^13b^

#### (2*R*,3*S*)-2-Methyl-6-oxo-3,6-dihydro-2*H*-pyran-3-yl acetate **(6**)

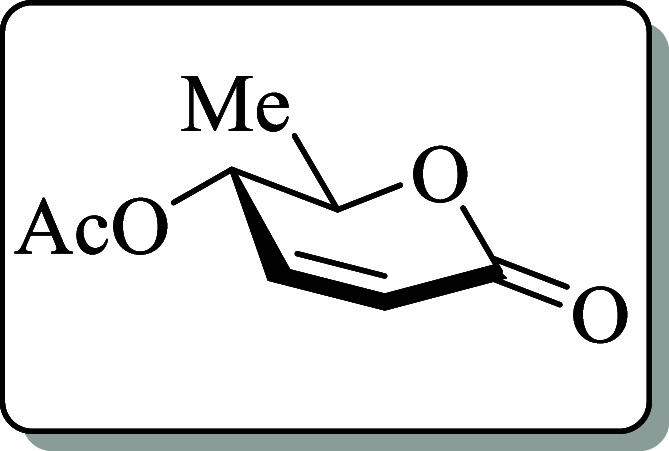



Compound **6** was obtained following the general procedure using **5** (20.0 mg, 0.093 mmol, 1.0 equiv) as starting material, NaClO_2_ (80% w/w, 15.7 mg, 0.140 mmol, 1.5 equiv) and TEMPO^+^ BF_4_^–^ (80.0 mg, 0.326 mmol, 3.5 equiv) in anhydrous acetonitrile (0.18 mL, [0.5 M]). The crude was purified by flash column chromatography on silica gel (hexane/ethyl acetate, 8:2) to obtain compound **6** (10.8 mg, 68% yield) as a colorless oil. R_f_ = 0.21 (silica gel, hexane/ethyl acetate, 8:2). ^**1**^**H NMR** (500 MHz, CDCl_3_) δ 6.76 (dd, *J* = 9.9, 3.4 Hz, 1H), 6.10 (dd, *J* = 9.9, 1.4 Hz, 1H), 5.26 (ddd, *J* = 7.0, 3.4, 1.5 Hz, 1H), 4.58 (q, *J* = 6.7 Hz, 1H), 2.13 (s, 3H), 1.42 (d, *J* = 6.6 Hz, 3H). ^**13**^**C{**^**1**^**H} NMR** (125 MHz, CDCl_3_) δ 170.1, 162.2, 142.9, 123.0, 76.6, 67.9, 20.9, 18.4. Spectroscopic data agree with those previously reported.^23^

#### (2*R*,3*R*)-2-Methyl-6-oxo-3,6-dihydro-2*H*-pyran-3-yl acetate (**8**)

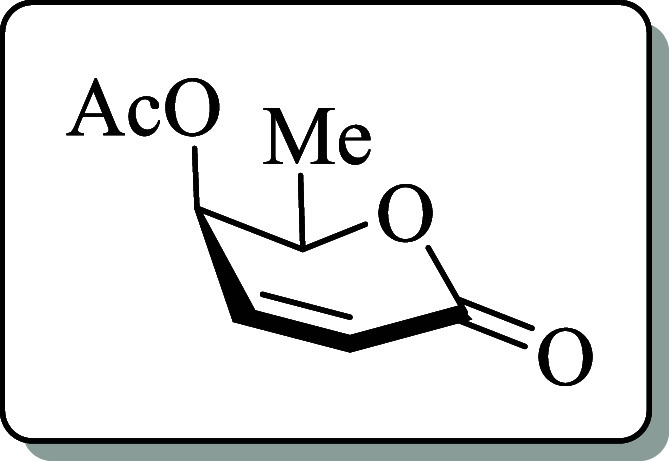



Compound **8** was obtained following the general procedure using **7** (21.0 mg, 0.098 mmol, 1.0 equiv) as starting material, NaClO_2_ (80% w/w,16.6 mg, 0.147 mmol, 1.5 equiv) and TEMPO^+^ BF_4_^–^ (83.3 mg, 0.343 mmol, 3.5 equiv) in anhydrous acetonitrile (0.19 mL, [0.5 M]). The crude was purified by flash column chromatography on silica gel (hexane/ethyl acetate, 8:2) to obtain compound **8** (11.2 mg, 67% yield) as a colorless oil. R_f_ = 0.16 (silica gel, hexane/ethyl acetate, 8:2). [α]_*D*_^25^ = −76.7 (c 0.073, CHCl_3_). ^**1**^**H NMR** (500 MHz, CDCl_3_) δ 6.96 (dd, *J* = 9.7, 5.8 Hz, 1H), 6.22 (d, *J* = 9.8 Hz, 1H), 5.18 (dt, *J* = 5.7, 2.2 Hz, 1H), 4.67 (qd, *J* = 6.6, 3.5 Hz, 1H), 2.13 (s, 3H), 1.43 (d, *J* = 6.7 Hz, 3H). ^**13**^**C{**^**1**^**H} NMR** (125 MHz, CDCl_3_) δ 170.2, 162.9, 140.3, 124.9, 75.1, 63.7, 20.5, 15.9. **HRMS-FAB***m*/*z*: [M + H]^+^ calcd for C_8_H_11_O_4_ 171.0657; found, 171.0672.

#### (*S*)-6-Oxo-3,6-dihydro-2*H*-pyran-3-yl acetate (**10**)

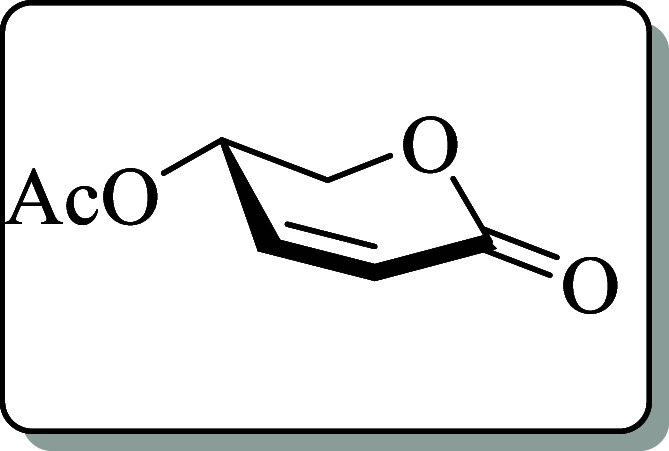



Compound **10** was obtained following the general procedure using **9** (20.0 mg, 0.10 mmol, 1.0 equiv) as starting material, NaClO_2_ (80% w/w,16.9 mg, 0.15 mmol, 1.5 equiv) and TEMPO^+^ BF_4_^–^ (85.0 mg, 0.35 mmol, 3.5 equiv) in anhydrous acetonitrile (0.20 mL, [0.5 M]). The crude was purified by flash column chromatography on silica gel (hexane/ethyl acetate, 9:1) to obtain compound **10** (10.6 mg, 68% yield) as a white solid. mp 91 °C. R_f_ = 0.38 (silica gel, hexane/ethyl acetate, 8:2). Compound **10** was also obtained following the general procedure using **11** (17.0 mg, 0.085 mmol) as starting material, NaClO_2_ (80% w/w, 14.4 mg, 0.127 mmol, 1.5 equiv) and TEMPO^+^ BF_4_^–^ (72.2 mg, 0.297 mmol, 3.5 equiv) in anhydrous acetonitrile (0.17 mL, [0.5 M]). The crude was purified by flash column chromatography on silica gel (hexane/ethyl acetate, 9:1) to obtain compound **10** (8.2 mg, 62% yield). [α]_*D*_^25^ = +137.1 (c 0.633, CHCl_3_).^**1**^**H NMR** (500 MHz, CDCl_3_) δ 6.92 (dd, *J* = 9.8, 5.0 Hz, 1H), 6.19 (d, *J* = 9.8 Hz, 1H), 5.33–5.30 (m, 1H), 4.53 (dd, *J* = 12.7, 3.7 Hz, 1H), 4.49 (dd, *J* = 12.7, 3.6 Hz, 1H), 2.11 (s, 3H). ^**13**^**C{**^**1**^**H} NMR** (125 MHz, CDCl_3_) δ 170.2, 162.0, 141.0, 124.8, 69.2, 62.1, 20.8. **HRMS-FAB***m*/*z*: [M + H]^+^ calcd for C_7_H_9_O_4_ 157.0501; found, 157.0504.

#### (2*R*,3*S*)-6-Oxo-2-((tosyloxy)methyl)-3,6-dihydro-2*H*-pyran-3-yl acetate (**13**)

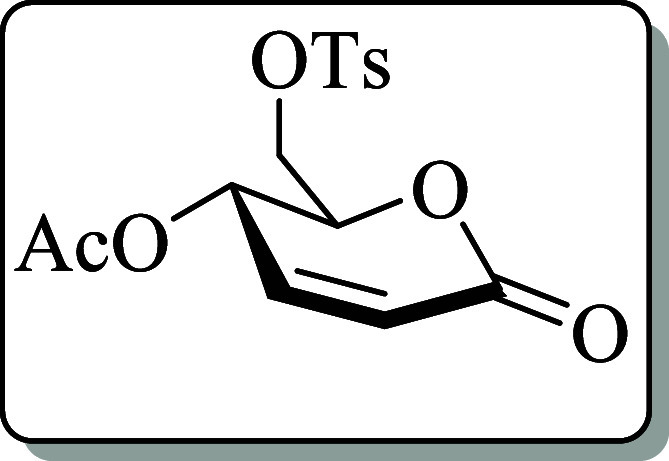



Compound **13** was obtained following the general procedure using **12** (30.0 mg, 0.078 mmol, 1.0 equiv) as starting material, NaClO_2_ (80% w/w, 13.2 mg, 0.117 mmol, 1.5 equiv) and TEMPO^+^ BF_4_^–^ (66.4 mg, 0.273 mmol, 3.5 equiv) in anhydrous acetonitrile (0.15 mL, [0.5 M]). The crude was purified by flash column chromatography on silica gel (hexane/ethyl acetate, 8:2) to obtain compound **13** (15.1 mg, 57% yield) as a colorless oil. R_f_ = 0.22 (silica gel, hexane/ethyl acetate, 7:3). ^**1**^**H NMR** (500 MHz, CDCl_3_) δ 7.79 (d, *J* = 8.0 Hz, 2H), 7.37 (d, *J* = 8.0 Hz, 2H), 6.78 (dd, *J* = 10.0, 3.0 Hz, 1H), 6.06 (d, *J* = 7.9, 1H), 5.53 – 5.51 (m, 1H), 4.65 – 4.61 (m, 1H), 4.28 (dd, *J* = 11.2, 4.1 Hz, 1H), 4.21 (dd, *J* = 11.3, 3.8 Hz, 1H), 2.46 (s, 3H), 2.11 (s, 3H). ^**13**^**C{**^**1**^**H} NMR** (125 MHz, CDCl_3_) δ 169.6, 160.6, 145.6, 143.4, 132.1, 130.1, 128.1, 122.2, 76.8, 66.8, 63.4, 21.7, 20.7. Spectroscopic data agree with those previously reported.^24^

#### (2*S*,3*S*)-2-(Iodomethyl)-6-oxo-3,6-dihydro-2*H*-pyran-3-yl acetate (**15**)

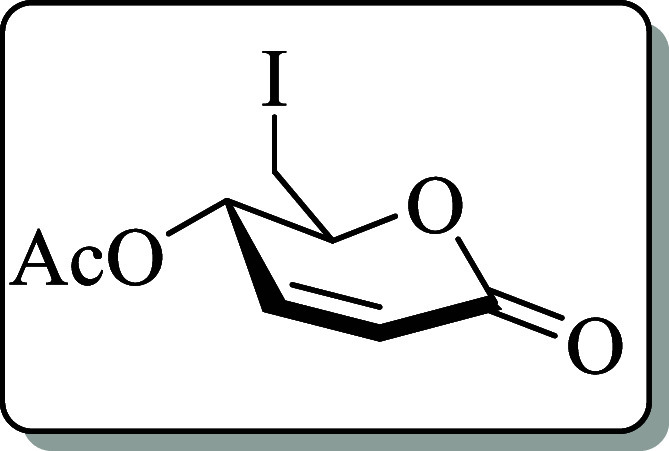



Compound **15** was obtained following the general procedure using **14** (20.0 mg, 0.058 mmol, 1.0 equiv) as starting material, NaClO_2_ (80% w/w, 9.8 mg, 0.087 mmol, 1.5 equiv) and TEMPO^+^ BF_4_^–^ (50.0 mg, 0.206 mmol, 3.5 equiv) in anhydrous acetonitrile (0.11 mL, [0.5 M]). The crude was purified by flash column chromatography on silica gel (hexane/ethyl acetate, 8:2) to obtain compound **15** (9.4 mg, 55% yield) as a colorless oil. R_f_ = 0.15 (silica gel, hexane/ethyl acetate, 8:2).[α]_*D*_^25^ = +75.1 (c 0.213, CHCl_3_). ^**1**^**H NMR** (500 MHz, CDCl_3_) δ 6.79 (dd, *J* = 9.9, 3.1 Hz, 1H), 6.11 (dd, *J* = 9.9, 1.7 Hz, 1H), 5.57 (ddt, *J* = 6.3, 3.0, 1.4 Hz, 1H), 4.43 (dt, *J* = 7.5, 5.1 Hz, 1H), 3.40 (qd, *J* = 5.9, 2.9 Hz, 2H), 2.16 (s, 3H).^**13**^**C{**^**1**^**H} NMR** (125 MHz, CDCl_3_) δ 169.7, 160.9, 143.0, 122.6, 78.4, 67.0, 20.9, 2.6. **HRMS-FAB***m*/*z*: [M – OAc]^+^ calcd for C_6_H_6_O_2_I 236.9413; found, 236.9416.

#### ((2*R*,3*S*)-3-(Benzoyloxy)-6-oxo-3,6-dihydro-2*H*-pyran-2-yl)methyl benzoate (**17**)

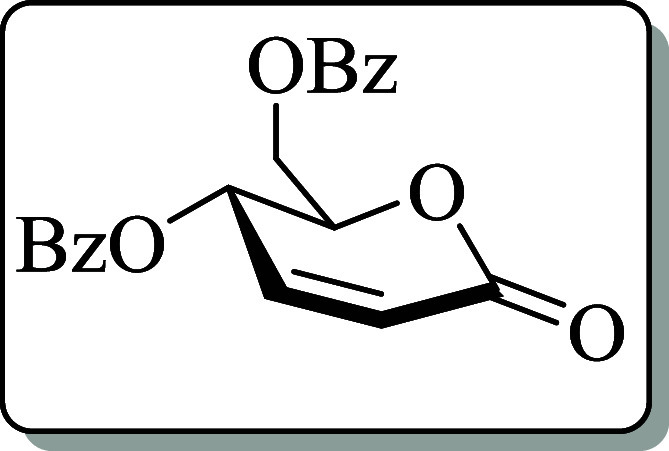



Compound **17** was obtained following the general procedure using **16** (45.0 mg, 0.098 mmol, 1.0 equiv) as starting material, NaClO_2_ (80% w/w, 16.6 mg, 0.147 mmol, 1.5 equiv) and TEMPO^+^ BF_4_^–^ (83.3 mg, 0.343 mmol, 3.5 equiv) in anhydrous acetonitrile (0.19 mL, [0.5 M]). The crude was purified by flash column chromatography on silica gel (hexane/ethyl acetate/DCM, 9:1:1) to obtain compound **17** (12.4 mg, 36% yield) as a colorless oil. R_f_ = 0.26 (silica gel, hexane/ethyl acetate, 8:2). ^**1**^**H NMR** (500 MHz, CDCl_3_) δ 8.06 – 7.98 (m, 4H), 7.65 – 7.54 (m, 2H), 7.48 – 7.41 (m, 4H), 6.95 (dd, *J* = 9.9, 3.3 Hz, 1H), 6.21 (dd, *J* = 9.9, 1.5 Hz, 1H), 5.89 (ddd, *J* = 7.2, 3.3, 1.6 Hz, 1H), 5.00 (m, 1H), 4.65 (apparent d, *J* = 4.4 H, 2H). ^**13**^**C{**^**1**^**H} NMR** (125 MHz, CDCl_3_) δ 166.1, 165.5, 161.3, 143.0, 134.1, 133.6, 130.1, 129.9, 129.3, 128.8, 128.7, 128.6, 123.0, 77.8, 64.3, 63.1. Spectroscopic data agree with those previously reported.^13b^

#### ((2*R*,3*R*)-3-(Benzoyloxy)-6-oxo-3,6-dihydro-2*H*-pyran-2-yl)methyl benzoate (**19**)

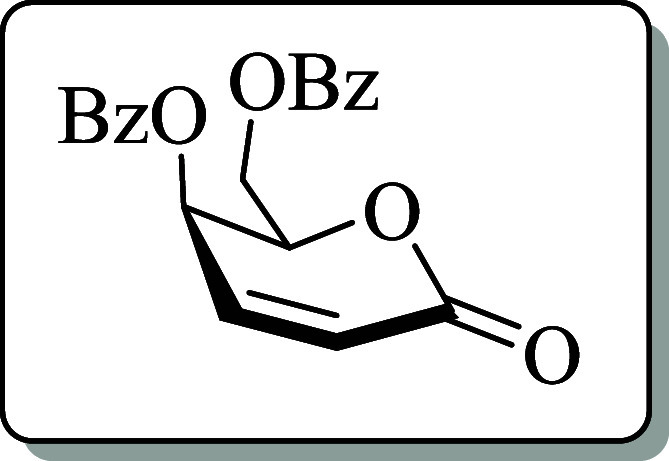



Compound **19** was obtained following the general procedure using **18** (45.8 mg, 0.1 mmol, 1.0 equiv) as starting material, NaClO_2_ (80% w/w, 16.9 mg, 0.150 mmol, 1.5 equiv) and TEMPO^+^ BF_4_^–^ (85.0 mg, 0.350 mmol, 3.5 equiv) in anhydrous acetonitrile (0.20 mL, [0.5 M]). The crude was purified by flash column chromatography on silica gel (hexane/ethyl acetate/DCM, 9:1:1) to obtain compound **19** (9.5 mg, 26% yield) as a colorless oil. R_f_ = 0.33 (silica gel, hexane/ethyl acetate, 7:3). [α]_*D*_^25^ = −136.0 (c 0.100, CHCl_3_) ^**1**^**H NMR** (500 MHz, CDCl_3_) δ 8.06 – 7.98 (m, 4H), 7.64 – 7.54 (m, 2H), 7.51 – 7.40 (m, 4H), 7.17 (ddd, *J* = 9.7, 5.8, 1.1 Hz, 1H), 6.32 (dd, *J* = 9.8, 1.2 Hz, 1H), 5.67 (ddd, *J* = 5.8, 2.8, 1.2 Hz, 1H), 5.07 – 5.00 (m, 1H), 4.76 – 4.60 (m, 2H). ^**13**^**C{**^**1**^**H} NMR** δ 166.2, 165.5, 161.8, 140.2, 137.5, 134.1, 133.6, 130.1, 130.0, 129.3, 128.8, 125.5, 76.5, 62.3, 62.2. **HRMS-FAB***m*/*z*: [M + H]^+^ calcd for C_20_H_17_O_6_ 353.1025; found, 353.1018.

#### (5*S*,6*R*)-5-(Benzyloxy)-6-((benzyloxy)methyl)-5,6-dihydro-2*H*-pyran-2-one (**21**)

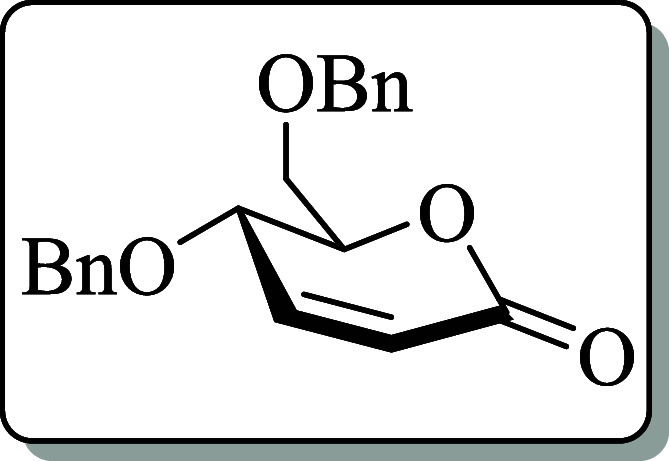



Compound **21** was obtained following the general procedure using **20** (26.0 mg, 0.062 mmol, 1.0 equiv) as starting material, NaClO_2_ (80% w/w, 10.6 mg, 0.093 mmol, 1.5 equiv) and TEMPO^+^ BF_4_^–^ (53.0 mg, 0.218 mmol, 3.5 equiv) in anhydrous acetonitrile (0.12 mL, [0.5 M]). The crude was purified by flash column chromatography on silica gel (hexane/ethyl acetate, 9:1) to obtain compound **21** (6.7 mg, 33.0% yield) as a colorless oil. R_f_ = 0.24 (silica gel, hexane/ethyl acetate, 8:2). ^**1**^**H NMR** (500 MHz, CDCl_3_) δ 7.38 – 7.24 (m, 10H), 6.84 (dd, *J* = 10.0, 2.2 Hz, 1H), 5.98 (dd, *J* = 10.1, 1.6 Hz, 1H), 4.67 – 4.43 (m, 6H), 3.80 (dd, *J* = 11.0, 3.2 Hz, 1H), 3.74 (dd, *J* = 11.0, 2.9 Hz, 1H). ^**13**^**C{**^**1**^**H} NMR** (125 MHz, CDCl_3_) δ 162.6, 146.3, 137.6, 137.0, 128.6, 128.5, 128.3, 128.1, 127.9, 127.8, 120.6, 80.1, 73.6, 72.3, 68.8, 67.9. Spectroscopic data agree with those previously reported.^14^

### Total Synthesis of (−)-Passifetilactone B and (−)-Passifetilactone C

#### (3a*R*,5a*R*,8a*S*,8b*R*)-2,2,7,7-Tetramethyltetrahydro-5*H*-bis([1,3]dioxolo)[4,5-*b*:4′,5′-*d*]pyran-5-carbaldehyde (**28**)

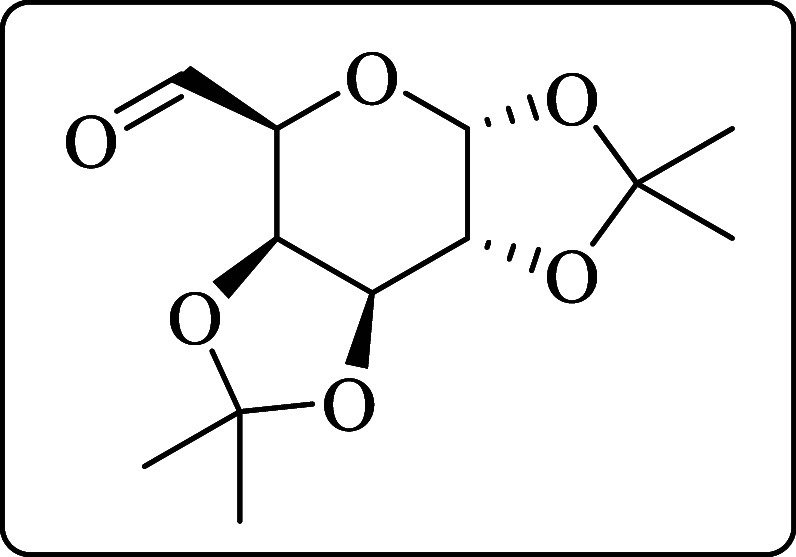



To a solution of 1,2:3,4-di-O-isopropylidene-α-D-galactopyranose (**27**) (1.0 g, 3.84 mmol, 1.0 equiv) in CH_2_Cl_2_ (5.22 mL) were added a saturated aqueous solution of NaHCO_3_ (35 mL), TEMPO (30.0 mg, 0.190 mmol, 0.05 equiv) and KBr (45.6 mg, 0.384 mmol, 0.1 equiv). The reaction mixture was cooled with an ice bath, and then a NaClO solution (13% chlorine, 5.22 mL) was added dropwise. After full consumption of the starting material (as judged by TLC), the reaction was quenched with a saturated aqueous solution of Na_2_S_2_O_3_. The aqueous phase was extracted with CH_2_Cl_2_ (3 × 20 mL), and the combined organic phase was dried with Na_2_SO_4_, filtered and the solvent evaporated under reduced pressure. The crude was purified by flash column chromatography on silica gel (hexane/ethyl acetate, gradient 9:1 to 7:3) to obtain compound **28** (0.994 g, 70% yield) as a yellow oil. R_f_ = 0.3 (silica gel, hexane/ethyl acetate, 7:3). [α]_*D*_^25^ = −124.5 (c = 0.760 in CHCl_3_) ^**1**^**H NMR** (500 MHz, CDCl_3_) δ 9.62 (s, 1H), 5.67 (d, *J* = 4.9 Hz, 1H), 4.65 (dd, *J* = 7.9, 2.5 Hz, 1H), 4.60 (dd, *J* = 7.8, 2.2 Hz, 1H), 4.39 (dd, *J* = 5.1, 2.5 Hz, 1H), 4.19 (d, *J* = 2.2 Hz, 1H), 1.51 (s, 3H), 1.44 (s, 3H), 1.35 (s, 3H), 1.32 (s, 3H). ^**13**^**C{**^**1**^**H} NMR** (125 MHz, CDCl_3_) δ 200.2, 110.0, 109.0, 96.3, 73.2, 71.7, 70.5, 70.4, 26.0, 25.8, 24.8, 24.3. **HRMS-EI** (*m*/*z*): [M – CH_3_]^+^ calcd for C_11_H_15_O_6_ 243.0867; found, 243.0869.

#### (3a*R*,5*R*,5a*S*,8a*S*,8b*R*)-2,2,7,7-Tetramethyl-5-((*Z*)-pentadec-1-en-1-yl)tetrahydro-5*H*-bis([1,3]dioxolo)[4,5-*b*:4′,5′-*d*]pyran (**29**)

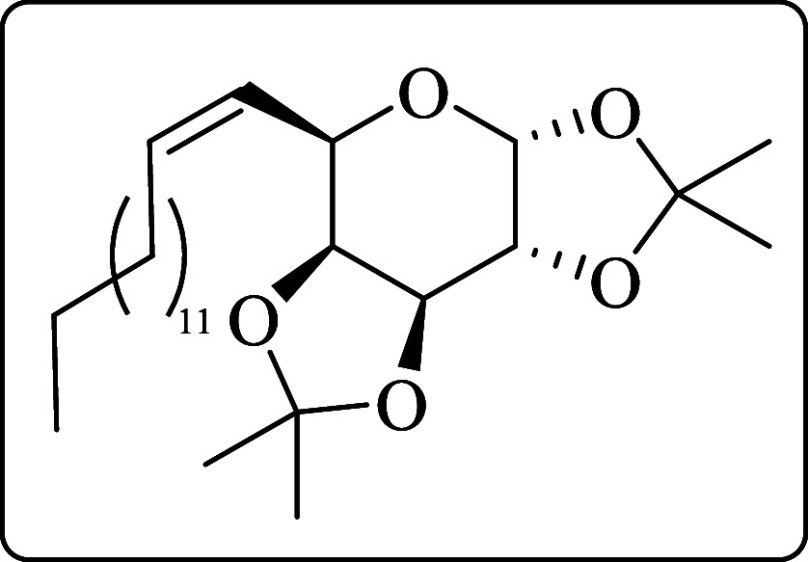



In a round-bottom flask, *n*-tetradecyl triphenylphosphonium bromide (2.87 g, 5.34 mmol, 2.0 equiv) was dissolved in anhydrous THF (15.0 mL) under a nitrogen atmosphere. Then, the mixture was cooled to −35 °C, and *n*-BuLi (3.42 mL, 2.5 M in *n*-hexane, 8.54 mmol, 3.2 equiv) was added dropwise over 15 min and stirred for an additional 1 h. After this time, aldehyde **28** (0.690 g, 2.67 mmol, 1.0 equiv) dissolved in anhydrous THF (3.0 mL) was added dropwise, and the reaction was stirred for 1 h at −35 °C. Then, the reaction mixture was allowed to warm to room temperature and stirred for an additional 9 h. After full consumption of the starting material (as judged by TLC), the reaction was quenched at 0 °C with a saturated aqueous solution of NH_4_Cl (20 mL). The mixture was extracted with Et_2_O (3 × 25 mL), dried with Na_2_SO_4_, filtered, and the solvent evaporated under reduced pressure. The crude was purified by flash column chromatography on silica gel (hexane/ethyl acetate, gradient 1:0 to 98:2) to obtain compound **29** (0.6559 g, 56% yield, single diastereoisomer) as a colorless oil. R_f_ = 0.44 (silica gel, hexane/ethyl acetate, 98:2). [α]_*D*_^25^ = −42.7 (c = 2.100 in CHCl_3_). ^**1**^**H NMR** (500 MHz, CDCl_3_) δ 5.69 – 5.64 (m, 1H), 5.61 – 5.59 (m, 1H), 5.56 (d, *J* = 5.1 Hz, 1H), 4.62 – 4.59 (m, 2H), 4.31 (dd, *J* = 5.1, 2.4 Hz, 1H), 4.15 (dd, *J* = 7.9, 2.1 Hz, 1H), 2.16 – 2.04 (m, 2H), 1.57 (s, 3H), 1.48 (s, 3H), 1.41 – 1.36 (m, 2H), 1.35 (s, 6H), 1.25 (bsm, 20H), 0.88 (t, *J* = 6.8 Hz, 3H). ^**13**^**C{**^**1**^**H} NMR** (125 MHz, CDCl_3_) δ 134.8, 125.1, 109.3, 108.5, 96.7, 73.7, 71.1, 70.4, 63.9, 32.1, 29.8, 29.8, 29.7, 29.6, 29.5, 28.4, 26.3, 26.1, 25.1, 24.5, 22.8. **HRMS-EI** (*m*/*z*): [M – CH_3_]^+^ calcd for C_25_H_43_O_5_ 423.3105; found, 423.3110.

#### (2*R*,3*S*,4*R*)-2-Pentadecyl-3,4-dihydro-2*H*-pyran-3,4-diyl diacetate (**30**)

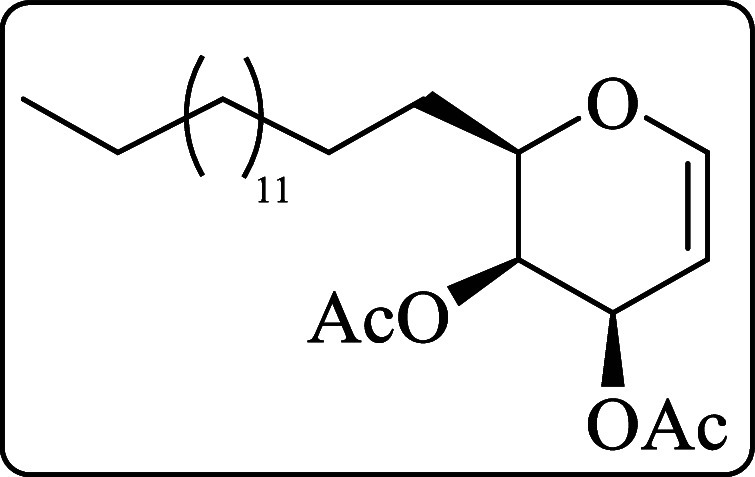



To a solution of compound **29** (0.452 g, 1.03 mmol, 1.0 equiv) in anhydrous EtOAc was added Pd/C (0.420 g, 10 wt %). Then, the mixture was stirred for 2 h at room temperature under a hydrogen atmosphere in a high-pressure autoclave reactor. Then, the reaction mixture was filtered over Celite to remove palladium on carbon and washed several times with EtOAc. The solvent was removed under reduced pressure to give a crude product, which was used in the next step without further purification.

To a stirred solution of the crude of the previous reaction in acetic acid (1.9 mL) at 0 °C, a mixture of acetic anhydride (0.48 mL) and concentrated H_2_SO_4_ (0.25 mL) was added dropwise. Then, the reaction was heated at 40 °C and stirred for 24 h. After this time, the reaction was quenched by pouring into ice water. Then, the aqueous layer was extracted with EtOAc (3 × 15 mL), and the combined organic phase was washed with aqueous NaHCO_3_, dried with Na_2_SO_4_, filtered and the solvent was evaporated under reduced pressure to give crude product, which was used in the next step without further purification.

A solution of the crude of the previous reaction in dry dichloromethane (2 mL) at 0 °C was added 33% (w/w) solution of hydrobromic acid in acetic acid (0.5 mL) and stirred for 2 h at room temperature. After the starting material was consumed (observed by TLC), the reaction mixture was diluted with dichloromethane (15 mL) and washed with ice-cold water (2 × 10 mL). Then, a solution of NaHCO_3_ was added and the aqueous phase was extracted with dichloromethane (3 × 20 mL), dried with Na_2_SO_4_, filtered, and the solvent was evaporated under reduced pressure to give crude product, which was used in the next step without further purification.

The crude of the previous reaction was dissolved in acetone (1.0 mL), and then a saturated sodium dihydrogen phosphate solution (2.0 mL) and zinc dust (0.416 g, 6.35 mmol, 12.5 equiv) were added. The reaction mixture was stirred for 2 h at room temperature. After this time, the mixture was filtered over Celite and the filtrate was extracted with EtOAc (3 × 15 mL). The organic phase was washed with water and saturated NaHCO_3_, dried with Na_2_SO_4_, filtered, and finally, the solvent was removed under reduced pressure. The crude was purified by flash column chromatography on silica gel (hexane/ethyl acetate, 95:5) to give compound **30** (0.169 g, 40% yield) as a white solid. mp 53–54 °C. R_f_ = 0.38 (silica gel, hexane/ethyl acetate, 95:5). [α]_*D*_^25^ = +0.4 (c 0.747, CHCl_3_). ^**1**^**H NMR** (500 MHz, CDCl_3_) δ 6.45 (dd, *J* = 6.3, 1.9 Hz, 1H), 5.57 – 5.52 (m, 1H), 5.32 (d, *J* = 4.6 Hz, 1H), 4.60 (dd, *J* = 6.3, 2.4 Hz, 1H), 3.98 (dd, *J* = 8.4, 5.0 Hz, 1H), 2.12 (s, 3H), 1.99 (s, 3H), 1.69 – 1.63 (m, 1H), 1.49 – 1.39 (m, 1H), 1.23 (bsm, 26H), 0.86 (t, *J* = 6.8 Hz, 3H). ^**13**^**C{**^**1**^**H} NMR** (125 MHz, CDCl_3_) δ 170.7, 170.4, 146.2, 98.5, 75.6, 65.5, 65.3, 32.0, 30.8, 29.79, 29.78, 29.75, 29.72, 29.62, 29.52, 29.46, 29.43, 25.1, 22.8, 20.9, 20.8, 14.2. **HRMS-FAB** (*m*/*z*): [M – OAc]^+^ calcd for C_22_H_39_O_3_ 351.2899; found, 351.2891.

#### (2*R*,3*R*)-6-Oxo-2-pentadecyl-3,6-dihydro-2*H*-pyran-3-yl acetate (**31**)

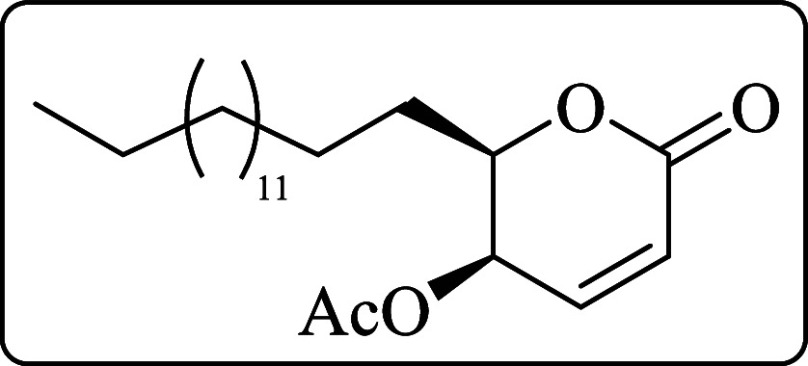



In a flame-dried sealed tube, compound **30** (30.0 mg, 0.073 mmol, 1.0 equiv), TEMPO^+^ BF_4_^–^ (62.1 mg, 0.255 mmol, 3.5 equiv) and sodium chlorite (80% w/w, 12.3 mg, 0.109 mmol, 1.5 equiv) were dissolved in anhydrous CH_3_CN (0.14 mL) under a nitrogen atmosphere. The reaction mixture was stirred and heated in an oil bath at 90 °C for 20 min. Upon completion of the reaction, the solvent was removed under vacuum, and the residue was purified on silica gel (hexane/ethyl acetate, 9:1) to obtain compound **31** (11.8 mg, 44% yield) as a white solid. mp 72–73 °C, R_f_ = 0.2 (silica gel, hexane/ethyl acetate, 9:1). [α]_*D*_^25^ = −137.7 (c 0.203, CHCl_3_). ^**1**^**H NMR** (500 MHz, CDCl_3_) δ 6.96 (dd, *J* = 9.6, 5.8 Hz, 1H), 6.21 (d, *J* = 9.6 Hz, 1H), 5.18 (dd, *J* = 5.9, 2.6 Hz, 1H), 4.45 (ddd, *J* = 8.4, 5.2, 2.6 Hz, 1H), 2.10 (s, 3H), 1.90 – 1.83 (m, 1H), 1.68 – 1.63 (m, 1H), 1.26 (brm, 26H), 0.88 (t, *J* = 6.8 Hz, 3H).^**13**^**C{**^**1**^**H} NMR** (125 MHz, CDCl_3_) δ 170.3, 163.1, 140.4, 125.3, 79.0, 63.2, 32.1, 30.2, 29.83, 29.80, 29.76, 29.66, 29.54, 29.51, 29.4, 25.0, 22.8, 20.7, 14.3. **HRMS-FAB***m*/*z*: [M – OAc]^+^ calcd for C_20_H_35_O_2_ 307.2637; found, 307.2639.

#### (−)-Passifetilactone **B**

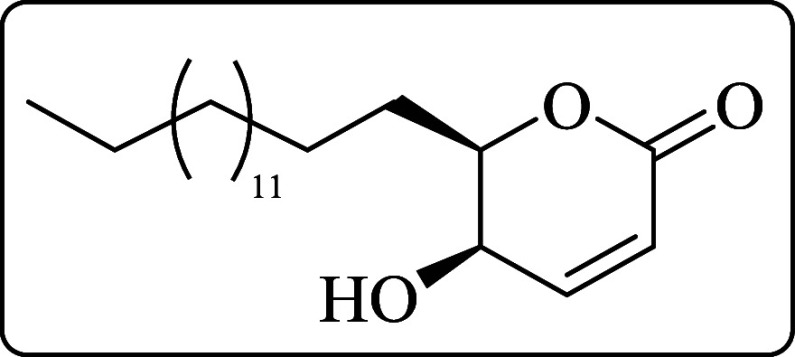



In a sealed tube contained compound **31** (8.0 mg, 0.021 mmol, 1.0 equiv), lipase acrylic resin (*Novozyme 435*) (100 mg c/mL *t*-BuOH), *t*-BuOH (0.1 mL) and H_2_O (4.0 equiv. 0.087 mmol, 1.56 μL) were stirred and heated in an oil bath at 45 °C for 8 h. Upon completion of the reaction, the solvent was removed under vacuum, and the residue was purified on silica gel (hexane/ethyl acetate, 7:3) to obtain **(−)-passifetilactone B** (5.8 mg, 85% yield) as a white solid. mp 71–72 °C, R_f_ = 0.22 (silica gel, hexane/ethyl acetate, 7:3). [α]_*D*_^25^ = −66.0 (c 0.333, MeOH). ^**1**^**H NMR** (500 MHz, CDCl_3_) δ 7.01 (dd, *J* = 9.7, 5.9 Hz, 1H), 6.11 (d, *J* = 9.6 Hz, 1H), 4.32 (ddd, *J* = 8.4, 6.0, 2.6 Hz, 1H), 4.07 (dd, *J* = 6.0, 2.6 Hz, 1H), 1.98 – 1.87 (m, 1H), 1.85 – 1.73 (m, 1H), 1.55 – 1.52 (m, 1H), 1.46 – 1.40 (m, 1H) 1.31 – 1.24 (brm, 24H), 0.88 (t, *J* = 6.8 Hz, 3H). ^**13**^**C{**^**1**^**H} NMR** (125 MHz, CDCl_3_) δ 163.8, 144.3, 123.3, 81.0, 62.3, 32.1, 30.2, 29.85, 29.81, 29.79, 29.71, 29.62, 29.58, 29.52, 25.1, 22.8, 14.3. **HRMS-EI** (*m*/*z*): [M]^+^ calcd for C_20_H_36_O_3_ 324.2668, found 324.2664.

#### (−)-Passifetilactone **C**

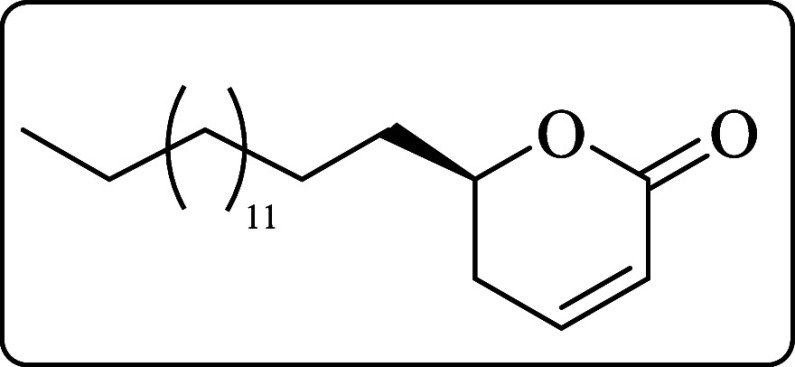



To a solution of compound **31** (6.5 mg, 0.017 mmol, 1.0 equiv) in THF (0.5 mL) was added zinc powder (10.8 mg, 0.17 mmol, 10.0 equiv) and the reaction was stirred for 5 min at room temperature. Then, a saturated aqueous solution of NH_4_Cl (0.5 mL) was added, and the mixture was stirred overnight at room temperature. Upon completion of the starting material (as judged by TLC), the reaction was filtered over Celite. Then, the filtrate was dried with Na_2_SO_4_, filtered and the solvent evaporated under reduced pressure to give crude product, which was used in the next step without further purification.

To a stirred solution of the crude of the previous reaction in anhydrous THF (0.5 mL), DBU (10 μL) was added and stirred for 2h at room temperature. Upon completion of the reaction (as judged by TLC), the solvent was removed under reduced pressure, and the residue was purified on silica gel (hexane/ethyl acetate, 9:1) to obtain **(−)-passifetilactone C** (3.7 mg, 71% yield) as a white solid. mp 57–58 °C, R_f_ = 0.37 (silica gel, hexane/ethyl acetate, 8:2). [α]_*D*_^25^ = −23.9 (c 0.353, MeOH). ^**1**^**H NMR** (500 MHz, CDCl_3_) δ 6.87 (m, 1H), 6.02 (dd, *J* = 9.6, 2.0 Hz, 1H), 4.41 (ddd, *J* = 9.7, 7.3, 5.0 Hz, 1H), 2.35 – 2.31 (m, 2H), 1.84 – 1.76 (m, 1H), 1.68 – 1.60 (m, 1H), 1.53 – 1.49 (m, 1H), 1.41 – 1.37 (m, 1H), 1.26 (brm, 24H), 0.87 (t, *J* = 7.05 Hz, 3H). ^**13**^**C{**^**1**^**H} NMR** (125 MHz, CDCl_3_) δ 164.8, 145.1, 121.6, 78.2, 35.0, 32.1, 29.84, 29.81, 29.78, 29.70, 29.62, 29.55, 29.52, 29.51, 25.0, 22.8, 14.3. **HRMS-FAB***m*/*z*: [M + H]^+^ calcd for C_20_H_37_O_2_ 309.2794; found, 309.2791.

## Data Availability

The data underlying this study are available in the published article and its Supporting Information.

## Supporting Information Available

^1^H and ^13^C spectra for new and relevant compounds (PDF)
