# Supplementary material for: Oxa-Ferrier Rearrangement Reaction Mediated by TEMPO Cation and NaClO2: Application to the Total Synthesis of Passifetilactones B and C
Source: J Org Chem. 2025 Apr 28;90(18):6251–60. doi: 10.1021/acs.joc.5c00354 (PMC12070465; doi:10.1021/acs.joc.5c00354)

*Supporting Information*

# Oxa-Ferrier Rearrangement Reaction Mediated by TEMPO Cation and $NaClO_2$: Application to the Total Synthesis of Passifetilactones B and C

*Jocelyn Bautista-Nava,[1] Luis F. Porras-Santos[1], Leticia Quintero,[1] José Alvano Pérez-Bautista,[1] Pedro López-Mendoza[*1] Fernando Sartillo-Piscil[1*]*

[1]Centro de Investigación de la Facultad de Ciencias Químicas, Benemérita Universidad Autónoma de Puebla (BUAP), 14 Sur Esq. San Claudio, Col. San Manuel, 72570, Puebla, México.

fernando.sartillo@correo.buap.mx; pedro.lopez@viep.com.mx

Fax: +52222 2454972; Tel: +52 222 2955500 ext. 7391.

# Table of Content

**1. Scaling the TEMPO$^+$-mediated oxa-Ferrier rearrangement**......S1

**2. Comparative tables for the NMR data of natural and synthetic passifetilactones B and C.**......S1

**3. $^1$H and $^{13}$C NMR spectra**......S4

$^1$H NMR spectrum of compound **2** (500 MHz, $CDCl_3$)......S4

$^{13}$C{1H} NMR spectrum of compound **2** (125 MHz, $CDCl_3$)......S5

$^1$H NMR spectrum of compound **4** (500 MHz, $CDCl_3$)......S6

$^{13}$C{1H} NMR spectrum of compound **4** (125 MHz, $CDCl_3$)......S7

$^1$H NMR spectrum of compound **6** (500 MHz, $CDCl_3$)......S8

$^{13}$C{$^1$H} NMR spectrum of compound **6** (125 MHz, $CDCl_3$)......S9

$^1$H NMR spectrum of compound **8** (500 MHz, $CDCl_3$)......S10

$^{13}$C{$^1$H} NMR spectrum of compound **8** (125 MHz, $CDCl_3$)......S11

$^1$H NMR spectrum of compound **10** (500 MHz, $CDCl_3$)......S12

$^{13}$C{$^1$H} NMR spectrum of compound **10** (125 MHz, $CDCl_3$)......S13

$^1$H NMR spectrum of compound **13** (500 MHz, CDCl3)......S14

$^{13}$C{$^1$H} NMR spectrum of compound **13** (125 MHz, $CDCl_3$)......S15

$^1$H NMR spectrum of compound **15** (500 MHz, $CDCl_3$)......S16

$^{13}$C{$^1$H} NMR spectrum of compound **15** (125 MHz, $CDCl_3$)......S17

$^1$H NMR spectrum of compound **17** (500 MHz, $CDCl_3$)......S18

$^{13}$C{$^1$H} NMR spectrum of compound **17** (125 MHz, $CDCl_3$)......S19

$^1$H NMR spectrum of compound **19** (500 MHz, $CDCl_3$)......S20

$^{13}$C{$^1$H} NMR spectrum of compound **19** (125 MHz, $CDCl_3$)......S21

$^1$H NMR spectrum of compound **21** (500 MHz, $CDCl_3$)......S22

$^{13}$C{$^1$H} NMR spectrum of compound **21** (125 MHz, $CDCl_3$)......S23

$^1$H NMR spectrum of compound **28** (500 MHz, $CDCl_3$)......S24

$^{13}$C{$^1$H} NMR spectrum of compound **28** (125 MHz, $CDCl_3$)......S25

$^1$H NMR spectrum of compound **29** (500 MHz, $CDCl_3$)......S26

$^{13}$C{$^1$H} NMR spectrum of compound **29** (125 MHz, $CDCl_3$)......S27

$^1$H NMR spectrum of compound **30** (500 MHz, $CDCl_3$)......S28

$^{13}C\{^1H\}$ NMR spectrum of compound **30** (125 MHz, $CDCl_3$) ........................................ S29
$^1H$ NMR spectrum of compound **31** (500 MHz, $CDCl_3$) ................................................ S30
$^{13}C\{^1H\}$ NMR spectrum of compound **31** (125 MHz, $CDCl_3$) ........................................ S31
$^1H$ NMR spectrum of **(-)-passifetilactone B** (500 MHz, $CDCl_3$) .................................... S32
$^{13}C\{^1H\}$ NMR spectrum of **(-)-passifetilactone B** (125 MHz, $CDCl_3$) ............................ S33
$^1H$ NMR spectrum of **(-)-passifetilactone C** (500 MHz, $CDCl_3$) .................................... S34
$^{13}C\{^1H\}$ NMR spectrum of **(-)-passifetilactone C** (125 MHz, $CDCl_3$) ............................ S35

## 1. Scaling the TEMPO$^+$-mediated oxa-Ferrier rearrangement

In a flame-dried tube, **1** (0.3403 g, 1.25 mmol, 1.0 equiv), TEMPO$^+$BF$_4^-$ (1.0639 g, 4.37 mmol, 3.5 equiv) and sodium chlorite (80%wt, 0.2120 g, 1.87 mmol, 1.5 equiv) were dissolved in anhydrous $CH_3CN$ (2.5 mL) under a nitrogen atmosphere. The reaction mixture was stirred and heated in an oil bath at 90° C for 20 minutes. Upon completion of the reaction, the solvent was removed under reduced pressure and the crude was purified by flash column chromatography on silica gel (hexane/ethyl acetate,7:3) to obtain compound **2** (0.1711g, 60% yield) as a colorless oil. $R_f$ = 0.23 (silica gel, hexane/ethyl acetate, 7:3).

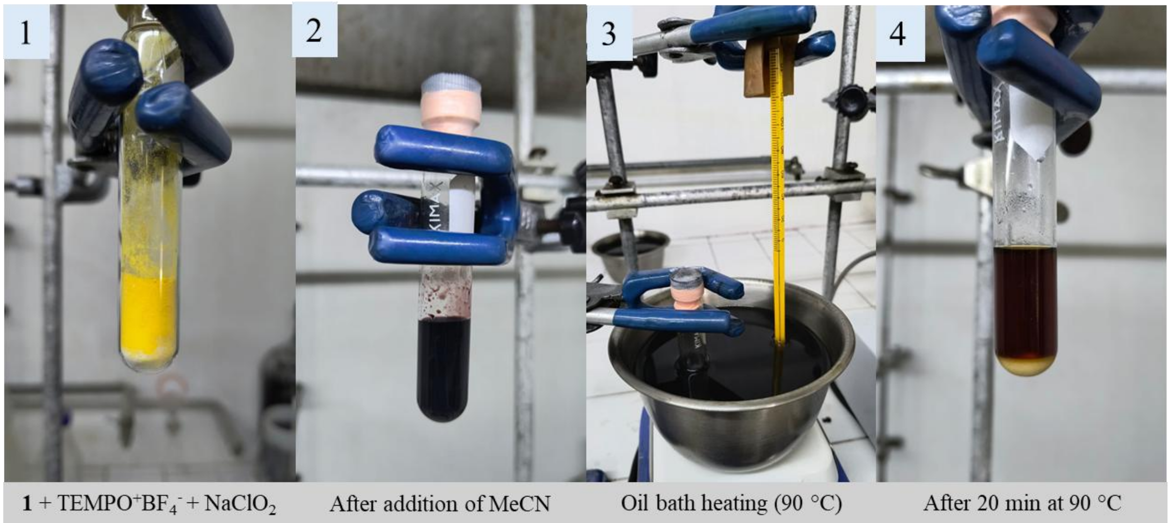


**Figure S1.** Scale-up process for the TEMPO+-mediated oxa-Ferrier rearrangement to compound **2**.

## 2. Comparative tables for the NMR data of natural and synthetic passifetilactones B and C.

**Table S1.** $^1$H NMR data for Passifetilactone B.

| Natural $^1$H (δ) | Synthetic $^1$H (δ) |
|---|---|
| 6.99 (dd, *J* = 9.6, 5.9 Hz, 1H) | 7.01 (dd, *J* = 9.7, 5.9 Hz, 1H) |
| 6.04 (d, *J* = 9.6 Hz, 1H) | 6.11 (d, *J* = 9.6 Hz, 1H) |
| 4.28 (ddd, *J* = 8.4,5.9, 2.5 Hz, 1H) | 4.32 (ddd, *J* = 8.4, 6.0, 2.6 Hz, 1H) |
| 4.02 (dd, *J* = 5.9, 2.6 Hz, 1H) | 4.07 (dd, *J* = 6.0, 2.6 Hz, 1H) |
| 1.89 (m, 1H) | 1.92 (m, 1H) |
| 1.79 (m, 1H) | 1.79 (m, 1H) |
| 1.49 (m, 1H) | 1.53 (m, 1H) |
| 1.41 (m, 1H) | 1.42 (m, 1H) |
| 1.21-1.35 (brm, 24H) | 1.26 (m, 24H) |
| 0.86, (t, *J* = 6.8 Hz, 3H) | 0.88 (t, *J* = 6.8 Hz, 3H) |

**Table S2**. $^{13}C$ NMR data for Passifetilactone B.

| Natural $^{13}C$ (δ) | Synthetic $^{13}C$ (δ) |
|---|---|
| 164.7 | 163.8 |
| 144.9 | 144.3 |
| 122.7 | 123.3 |
| 81.3 | 81.0 |
| 62.0 | 62.3 |
| 32.0 | 32.1 |
| 30.1 | 30.2 |
| 29.8 | 29.8 |
| | 29.8 |
| 29.8 | 29.8 |
| 29.7 | 29.7 |
| 29.6 | 29.6 |
| 29.6 | 29.6 |
| 29.6 | 29.5 |
| 25.1 | 25.1 |
| 22.8 | 22.8 |
| 14.2 | 14.3 |

**Table S3**. $^{1}H$ NMR data for Passifetilactone C.

| Natural $^{1}H$ (δ) | Synthetic $^{1}H$ (δ) |
|---|---|
| 6.87 (m, 1H) | 6.87 (m, 1H) |
| 6.01 (ddd, *J* = 9.8, 2.2, 1.5 Hz, 1H) | 6.02 (dd, *J* = 9.6, 2.0 Hz, 1H) |
| 4.41 (ddt, *J* = 10.1, 7.3, 5.4 Hz, 1H) | 4.41 (ddd, *J* = 9.7, 7.3, 5.0 Hz, 1H) |
| 2.32 (m, 1H) | 2.35 – 2.31 (m, 2H) |
| 1.79 (m, 1H) | 1.84 – 1.76 (m, 1H) |
| 1.63 (m, 1H) | 1.68 – 1.60 (m, 1H) |
| 1.50 (m, 1H) | 1.53 – 1.49 (m, 1H) |
| 1.39 (m, 1H) | 1.41 – 1.37 (m, 1H) |
| 1.28 (brm, 24H) | 1.26 (brm, 24H) |
| 0.87 (t, *J* = 6.8 Hz, 3H) | 0.87 (t, *J* = 7.05 Hz, 3H). |

**Table S4**. $^{13}$C NMR data for Passifetilactone C.

| **Natural** $^{13}$**C (δ)** | **Synthetic** $^{13}$**C (δ)** |
|---|---|
| 164.8 | 164.8 |
| 145.2 | 145.1 |
| 121.6, | 121.6 |
| 78.2 | 78.2 |
| 35.0 | 35.0 |
| 32.1 | 32.1 |
| 29.8 | 29.8 |
| 29.8 | 29.8 |
| 29.8 | 29.8 |
| 29.7 | 29.7 |
| 29.6 | 29.6 |
| | 29.6 |
| 29.5 | 29.5 |
| 29.5 | 29.5 |
| 24.9 | 25.0 |
| 22.8 | 22.8 |
| 14.3 | 14.3 |

## 3. $^{1}$H and $^{13}$C NMR spectra

$^{1}$H NMR spectrum of compound **2** (500 MHz, $CDCl_3$)

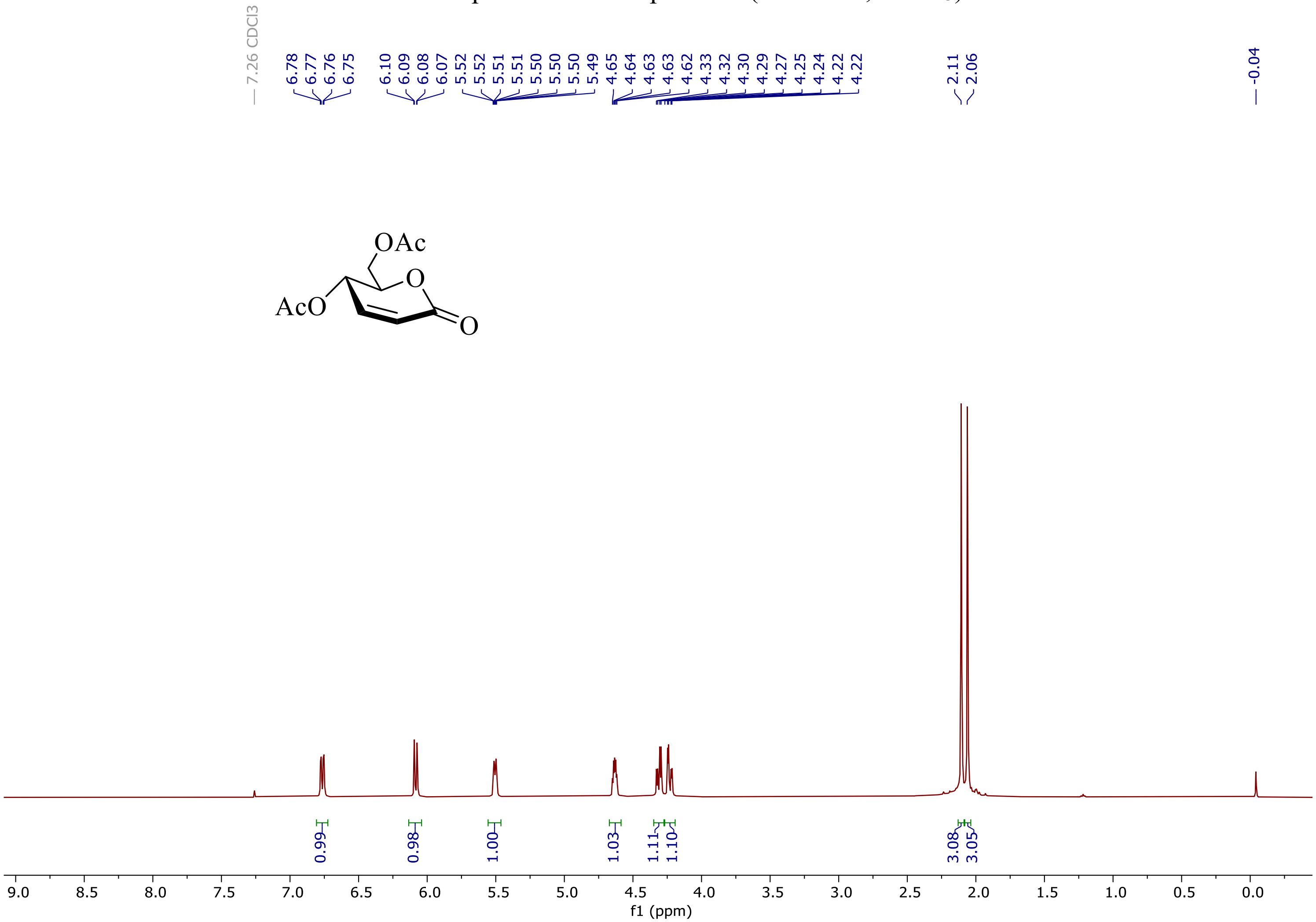

$^{13}$C{1H} NMR spectrum of compound **2** (125 MHz, $CDCl_3$)

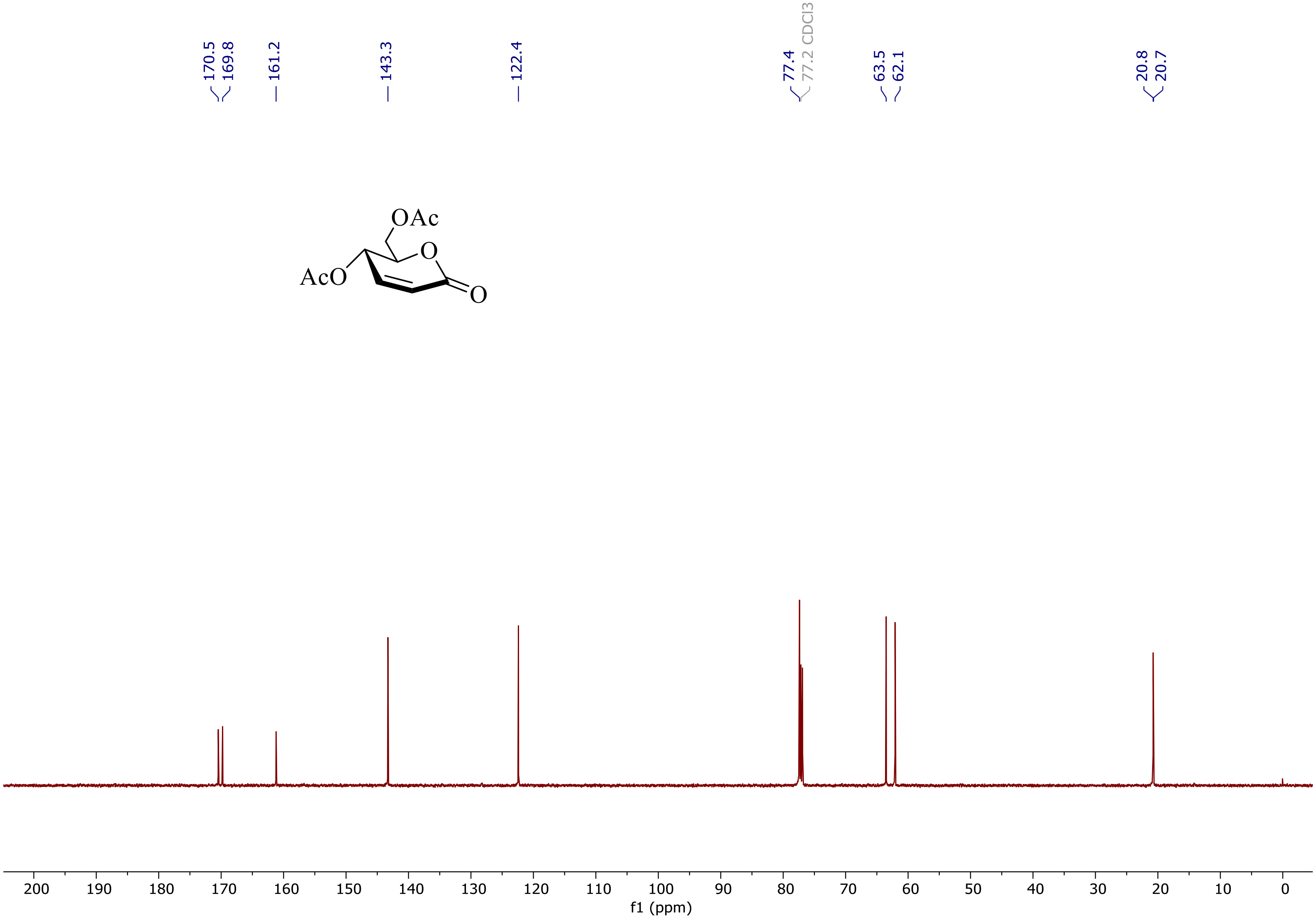

$^{1}$H NMR spectrum of compound **4** (500 MHz, $CDCl_3$)

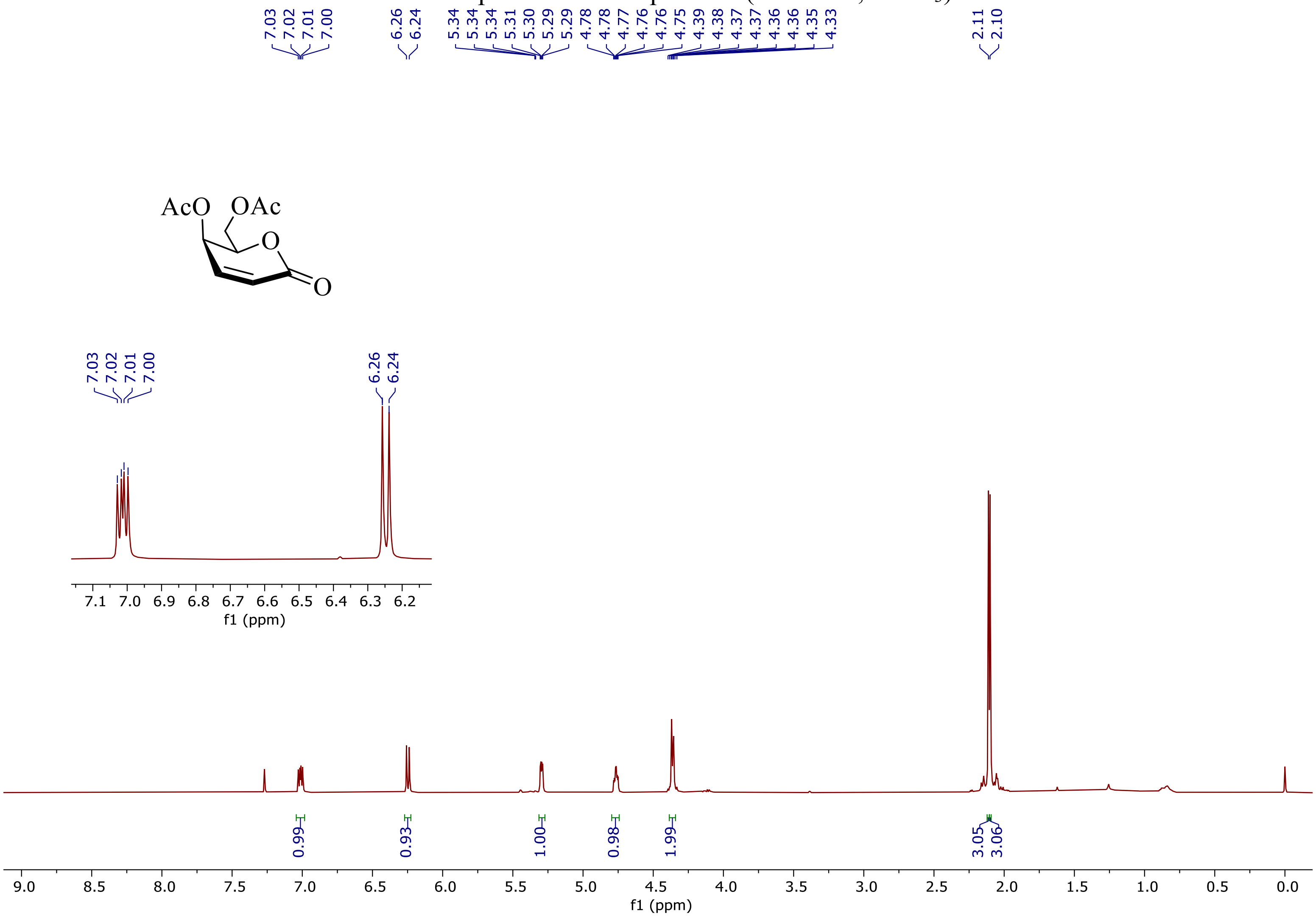

$^{13}C\{1H\}$ NMR spectrum of compound **4** (125 MHz, $CDCl_3$)

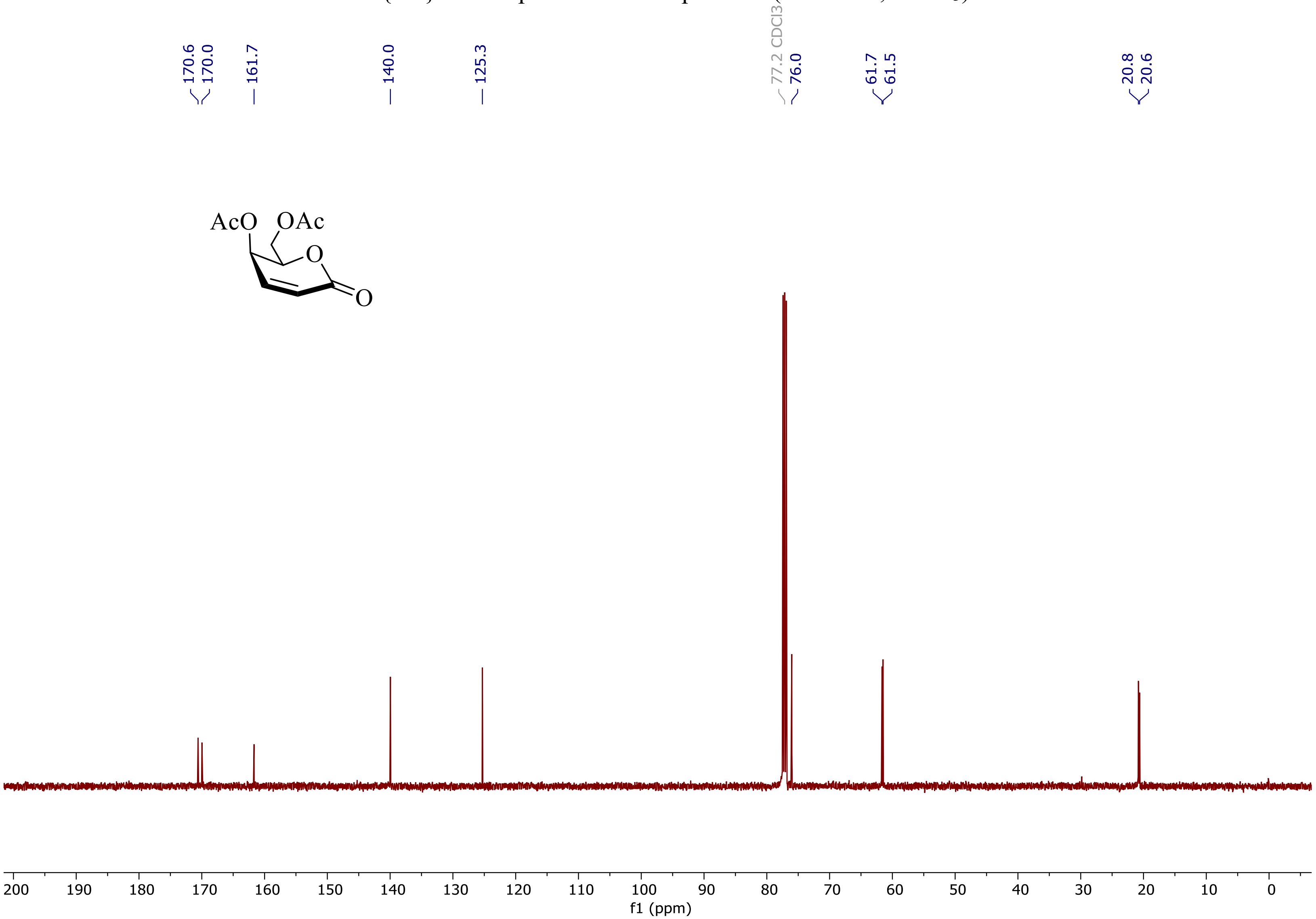

$^1$H NMR spectrum of compound **6** (500 MHz, $CDCl_3$)

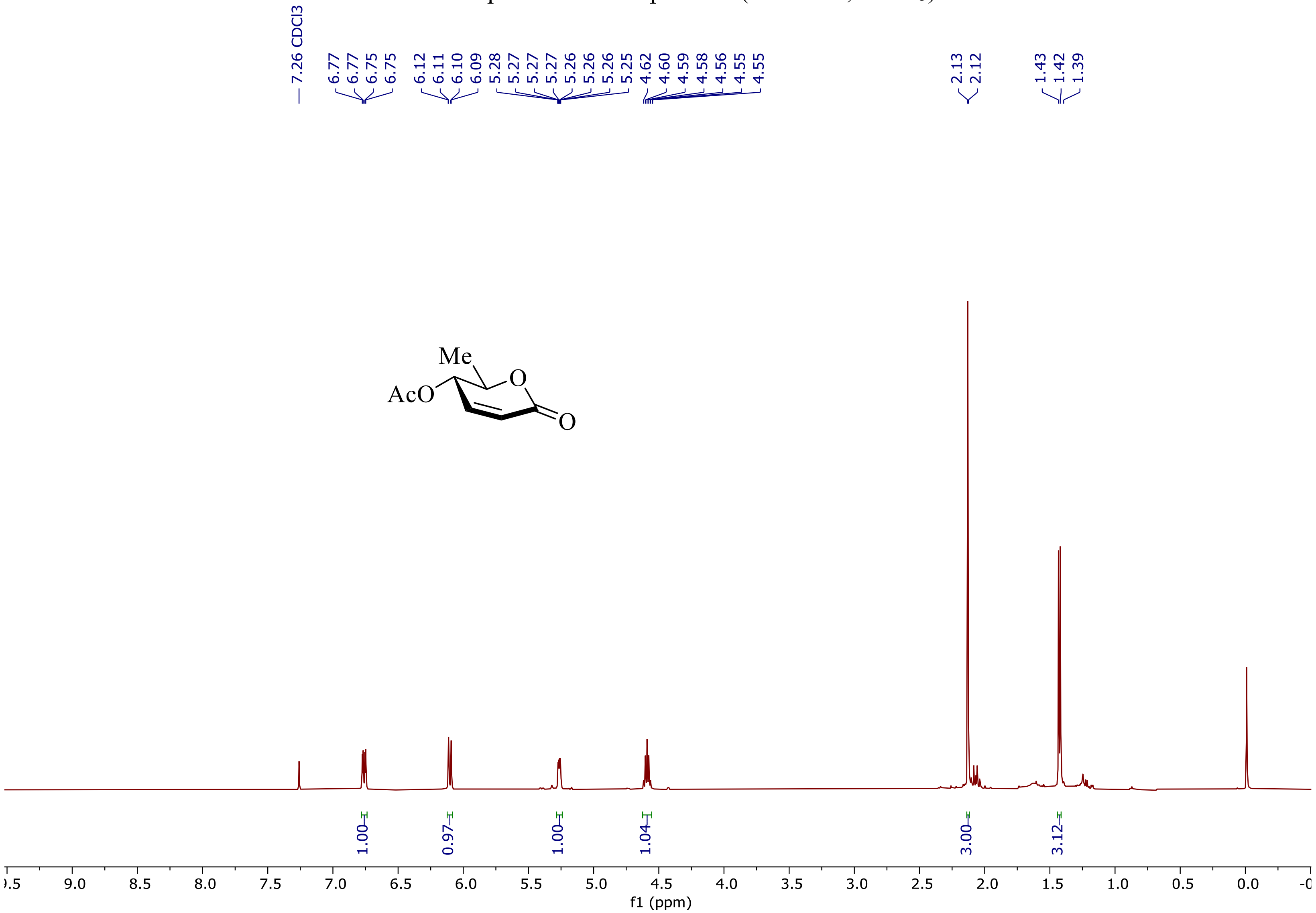

$^{13}C\{^1H\}$ NMR spectrum of compound **6** (125 MHz, $CDCl_3$)

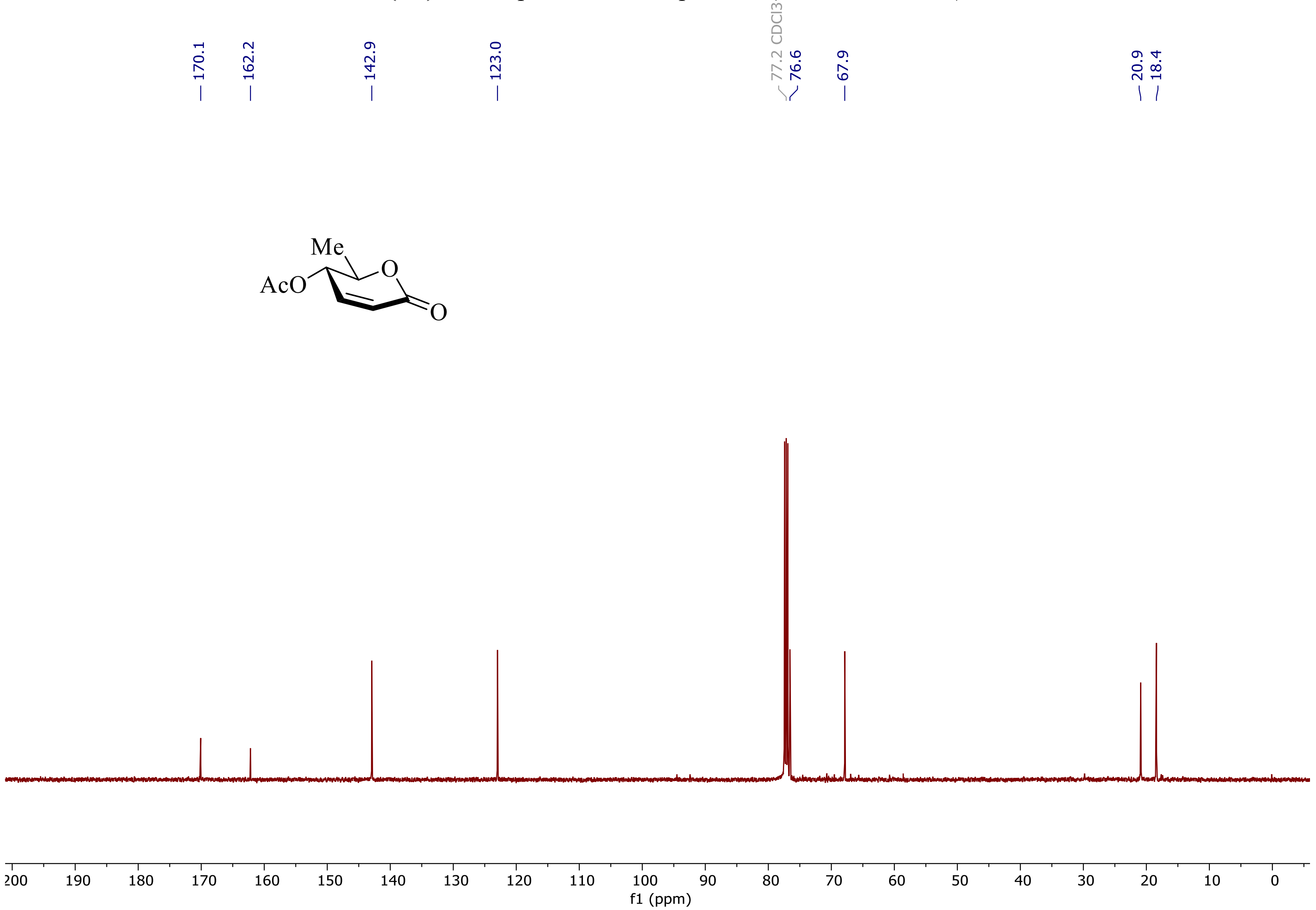

$^{1}$H NMR spectrum of compound **8** (500 MHz, $CDCl_3$)

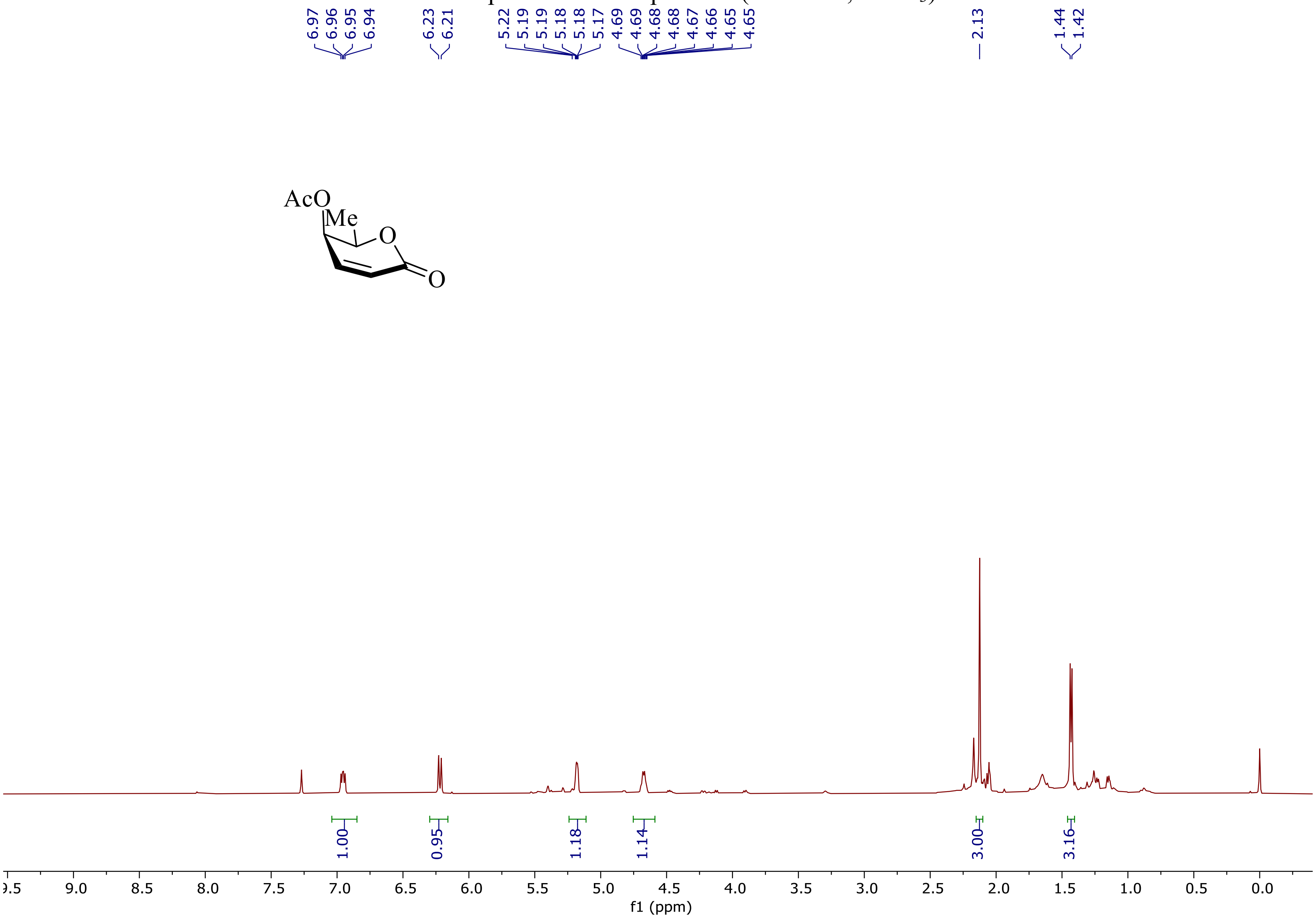

$^{13}C\{^{1}H\}$ NMR spectrum of compound **8** (125 MHz, $CDCl_3$)

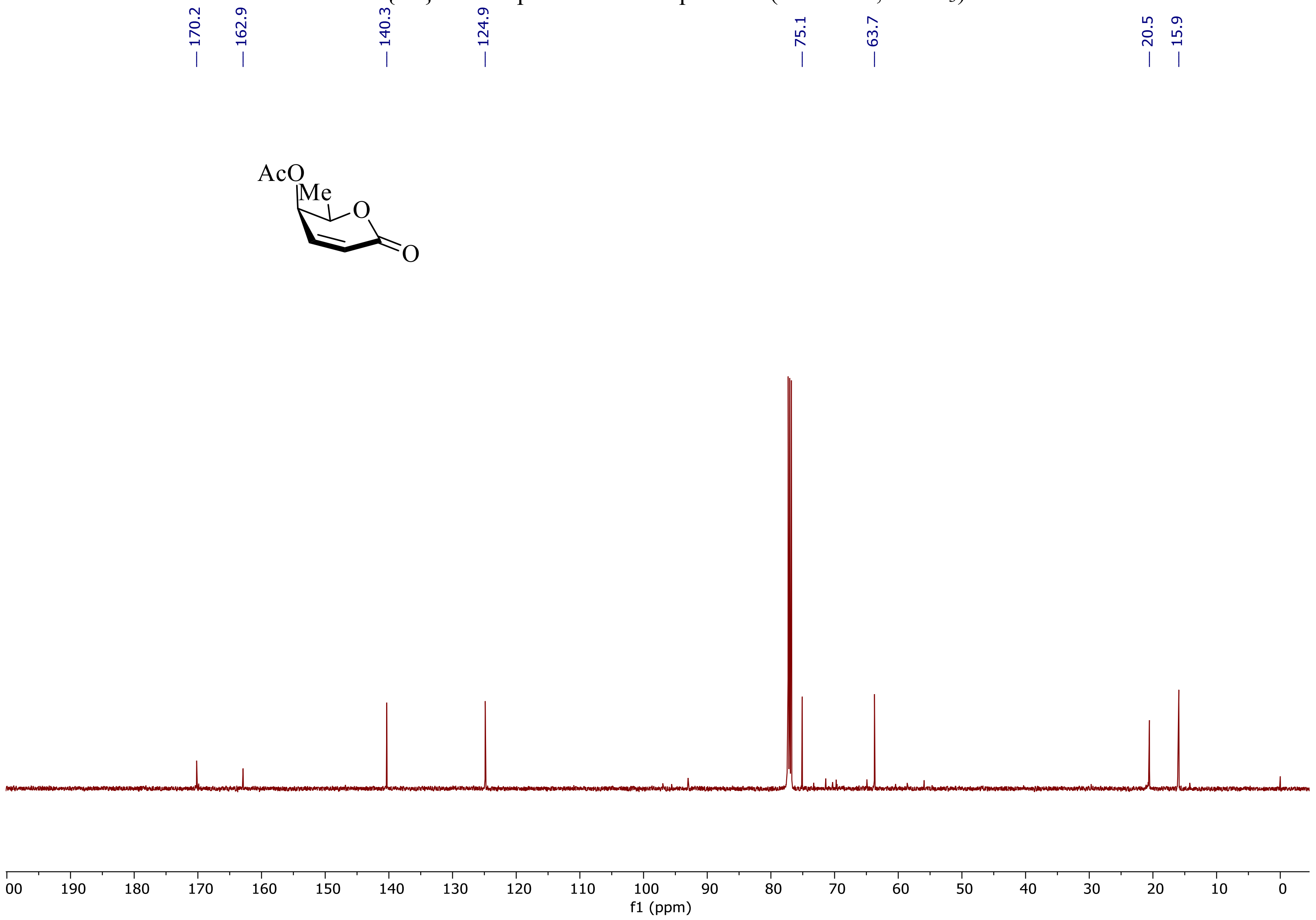

$^{1}$H NMR spectrum of compound **10** (500 MHz, $CDCl_3$)

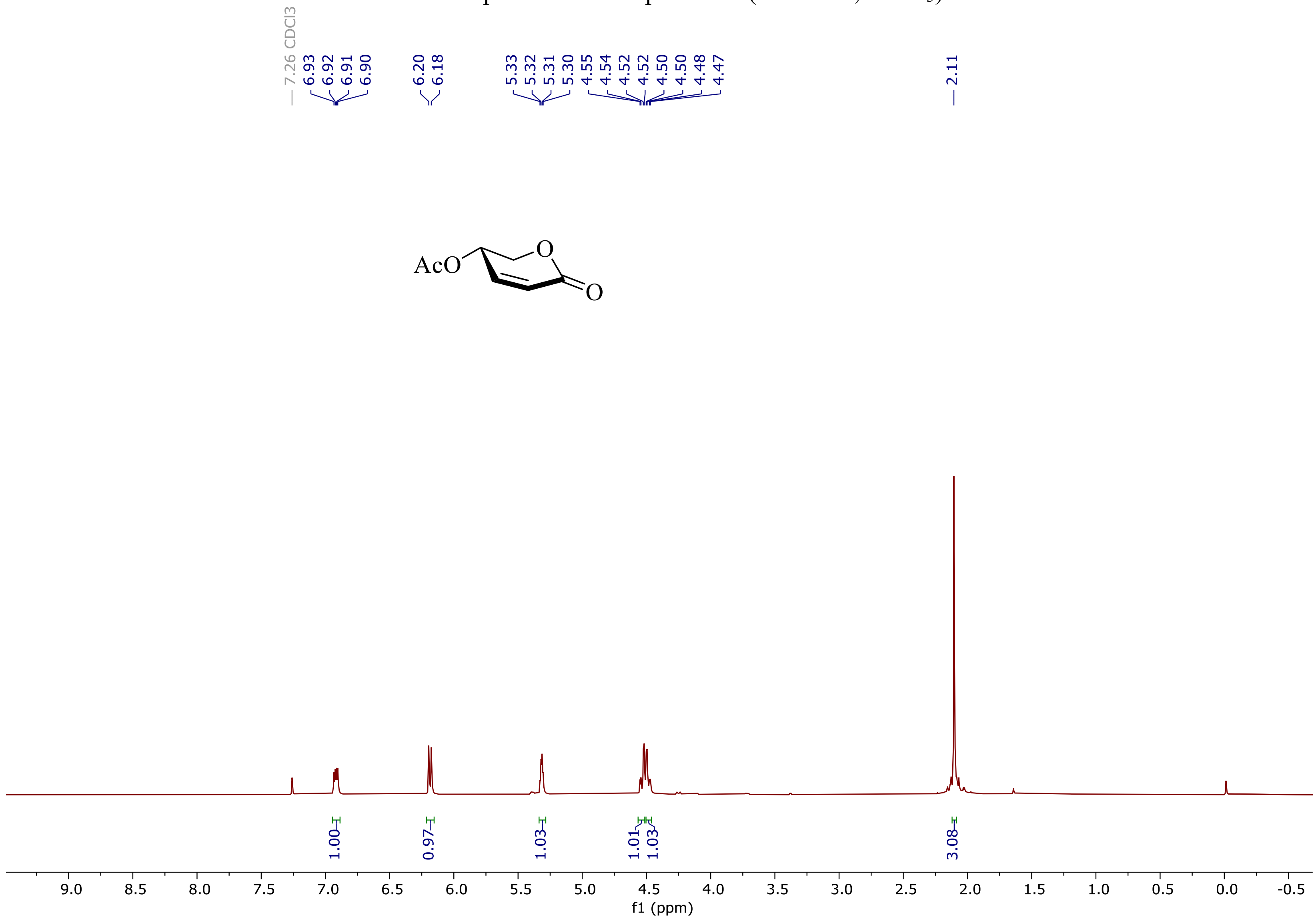

$^{13}C\{^{1}H\}$ NMR spectrum of compound **10** (125 MHz, $CDCl_3$)

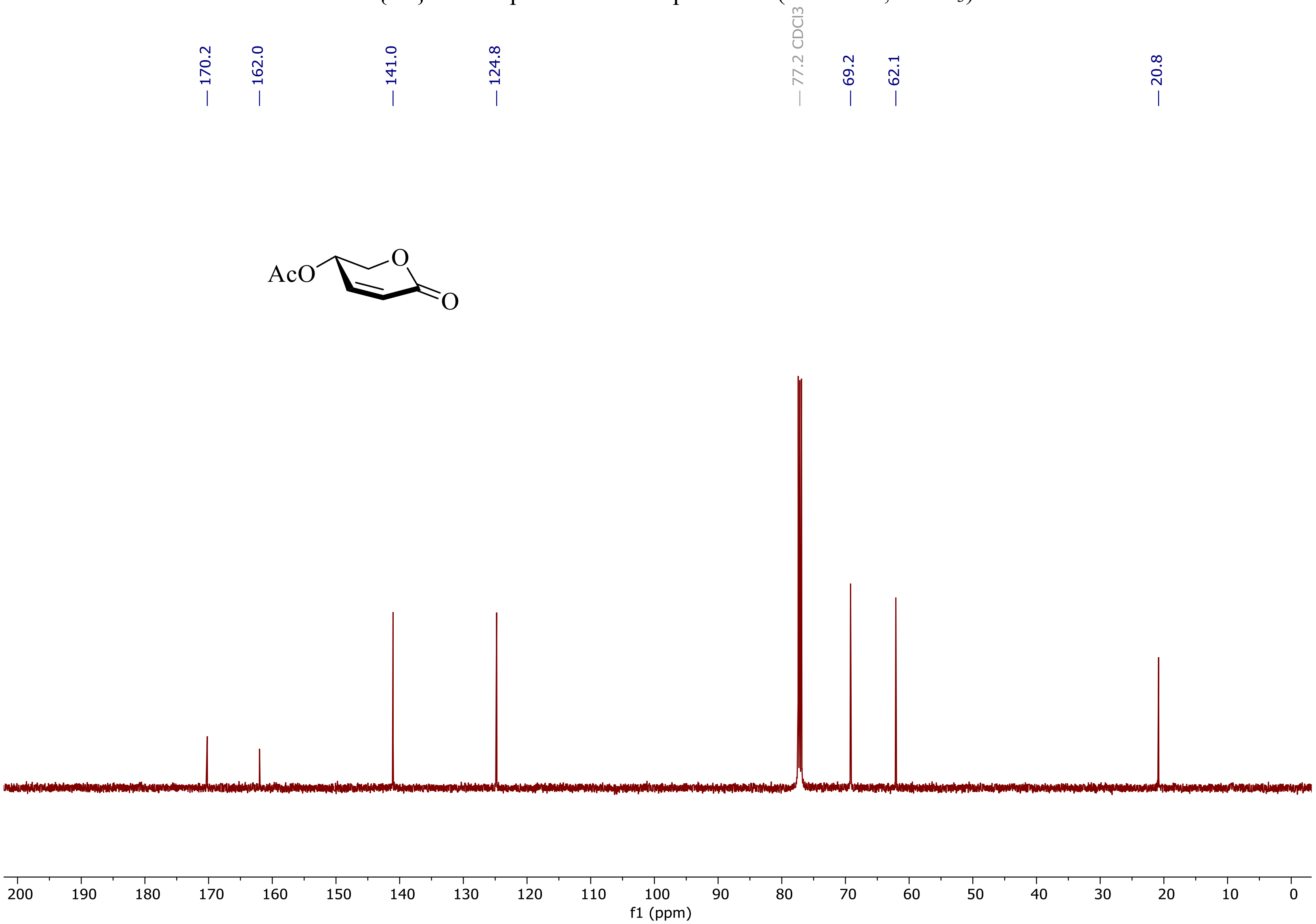

$^{1}$H NMR spectrum of compound **13** (500 MHz, CDCl3)

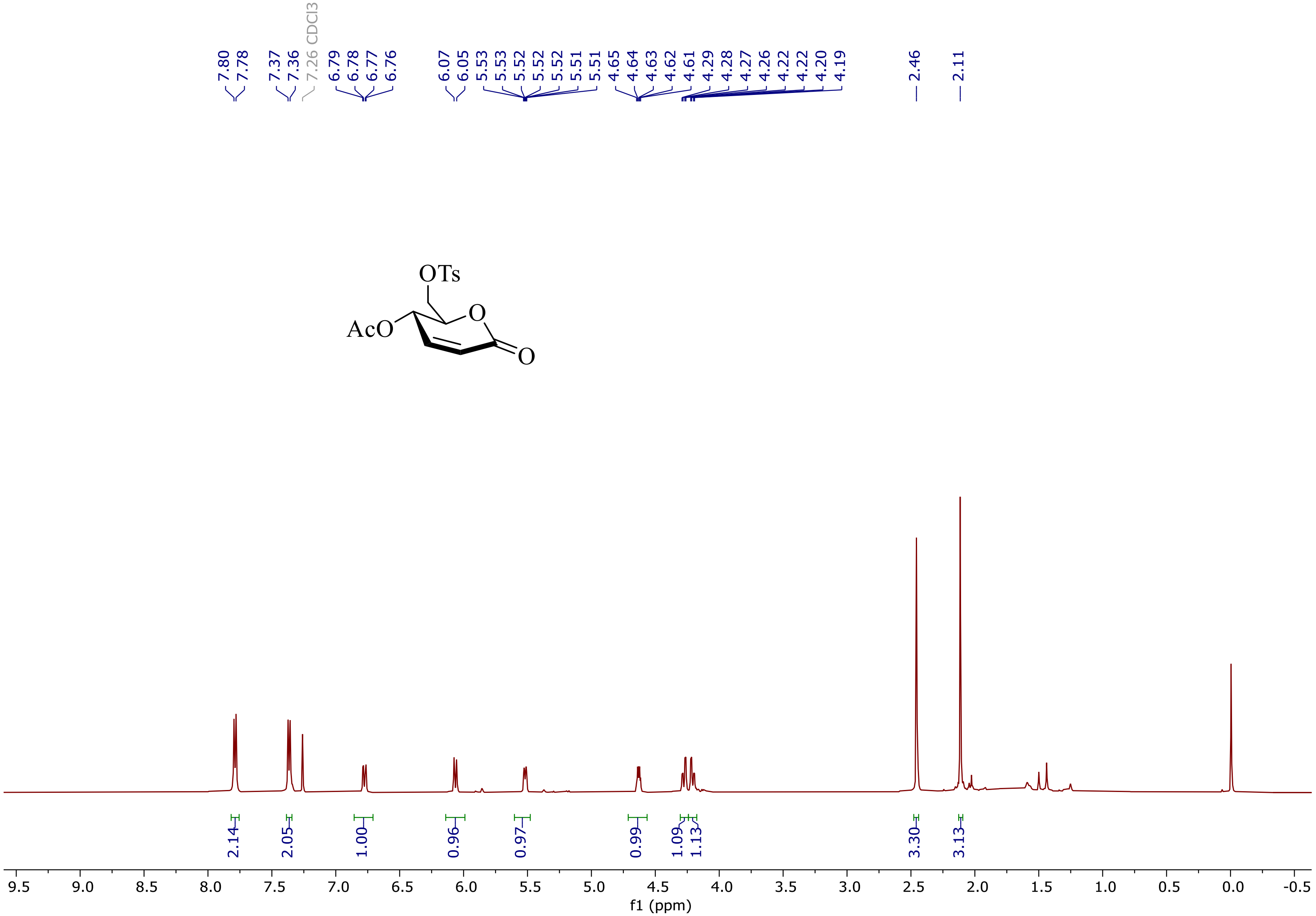

$^{13}C\{^{1}H\}$ NMR spectrum of compound **13** (125 MHz, $CDCl_3$)

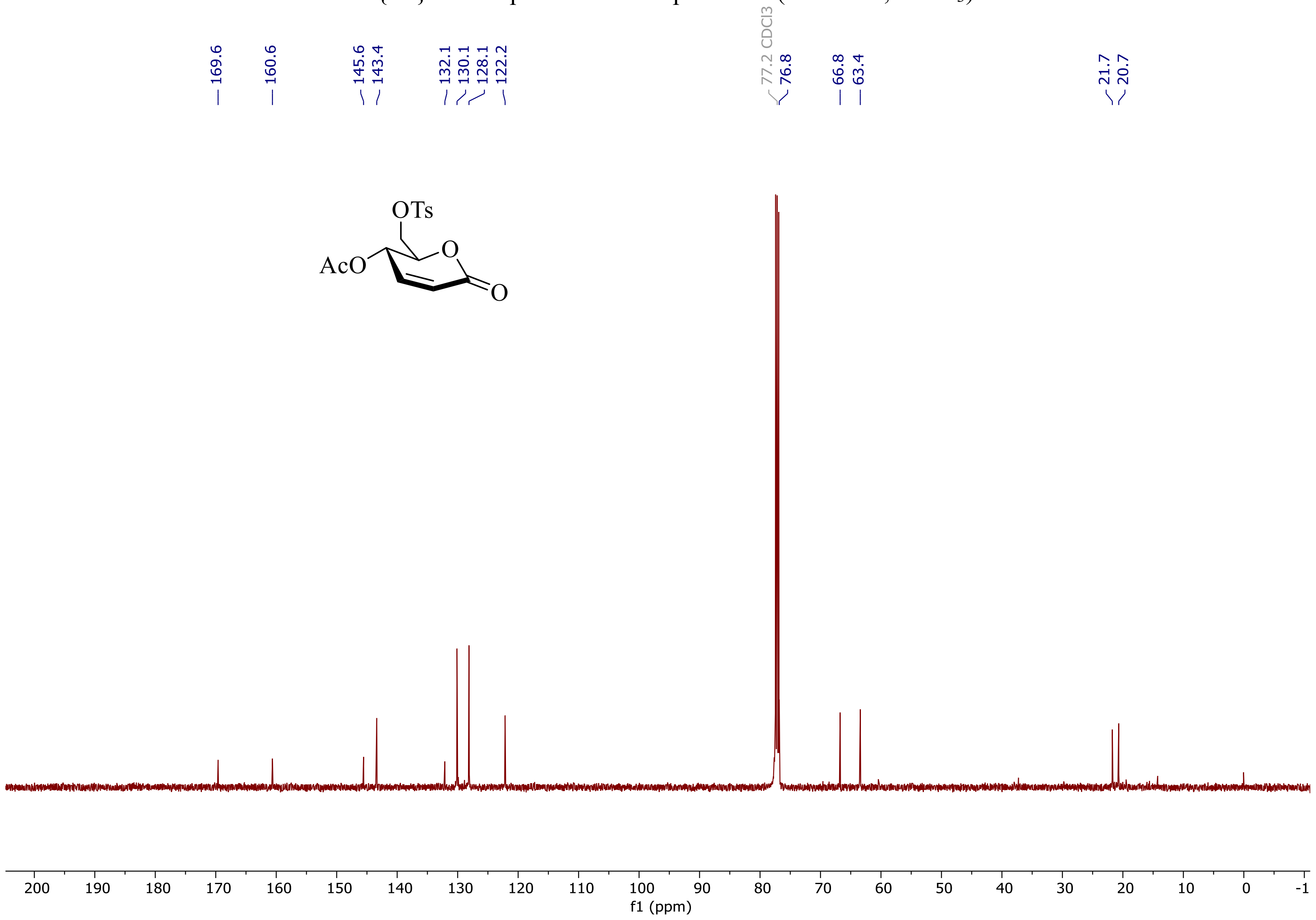

$^1$H NMR spectrum of compound **15** (500 MHz, $CDCl_3$)

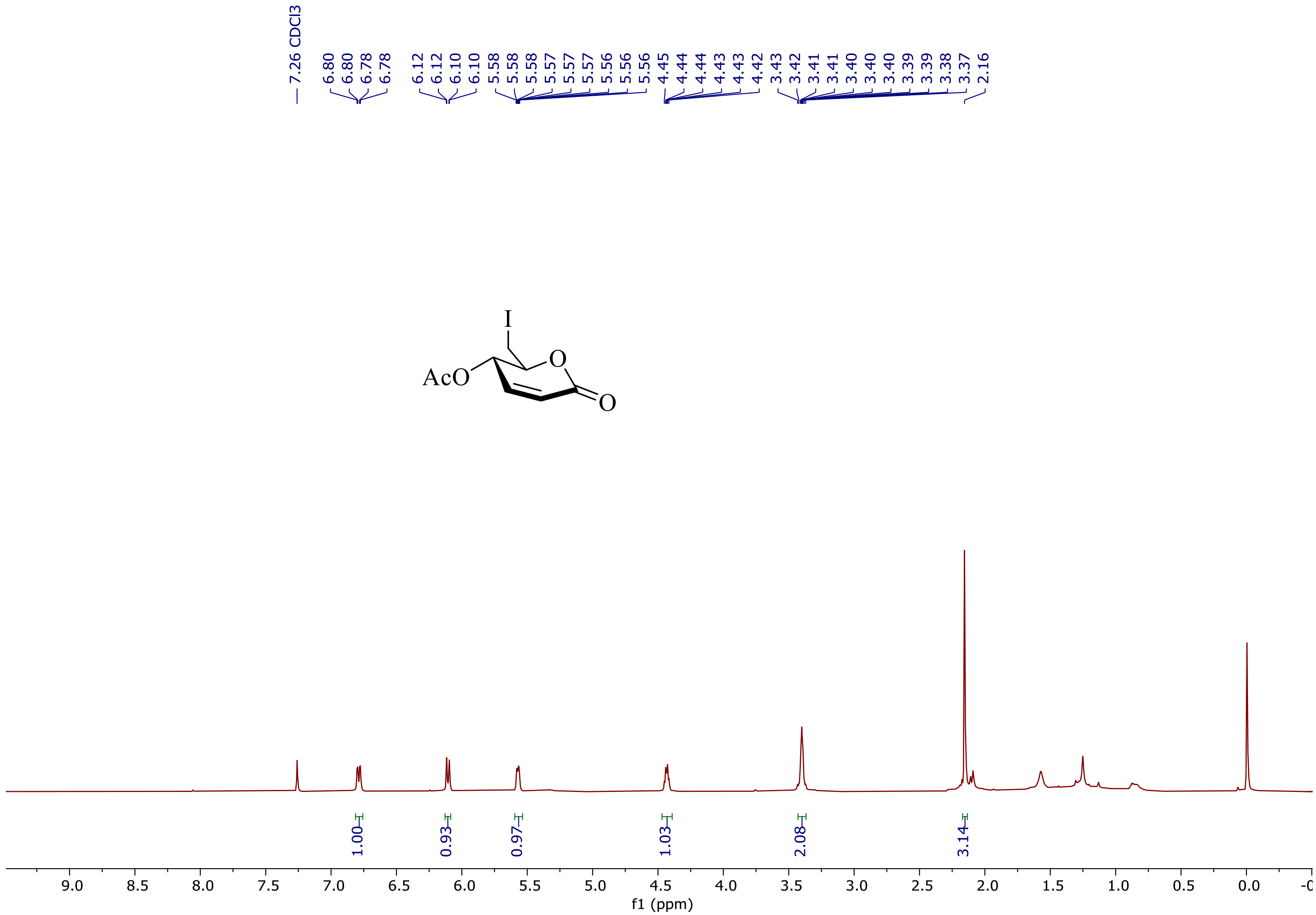

$^{13}C\{^{1}H\}$ NMR spectrum of compound **15** (125 MHz, $CDCl_3$)

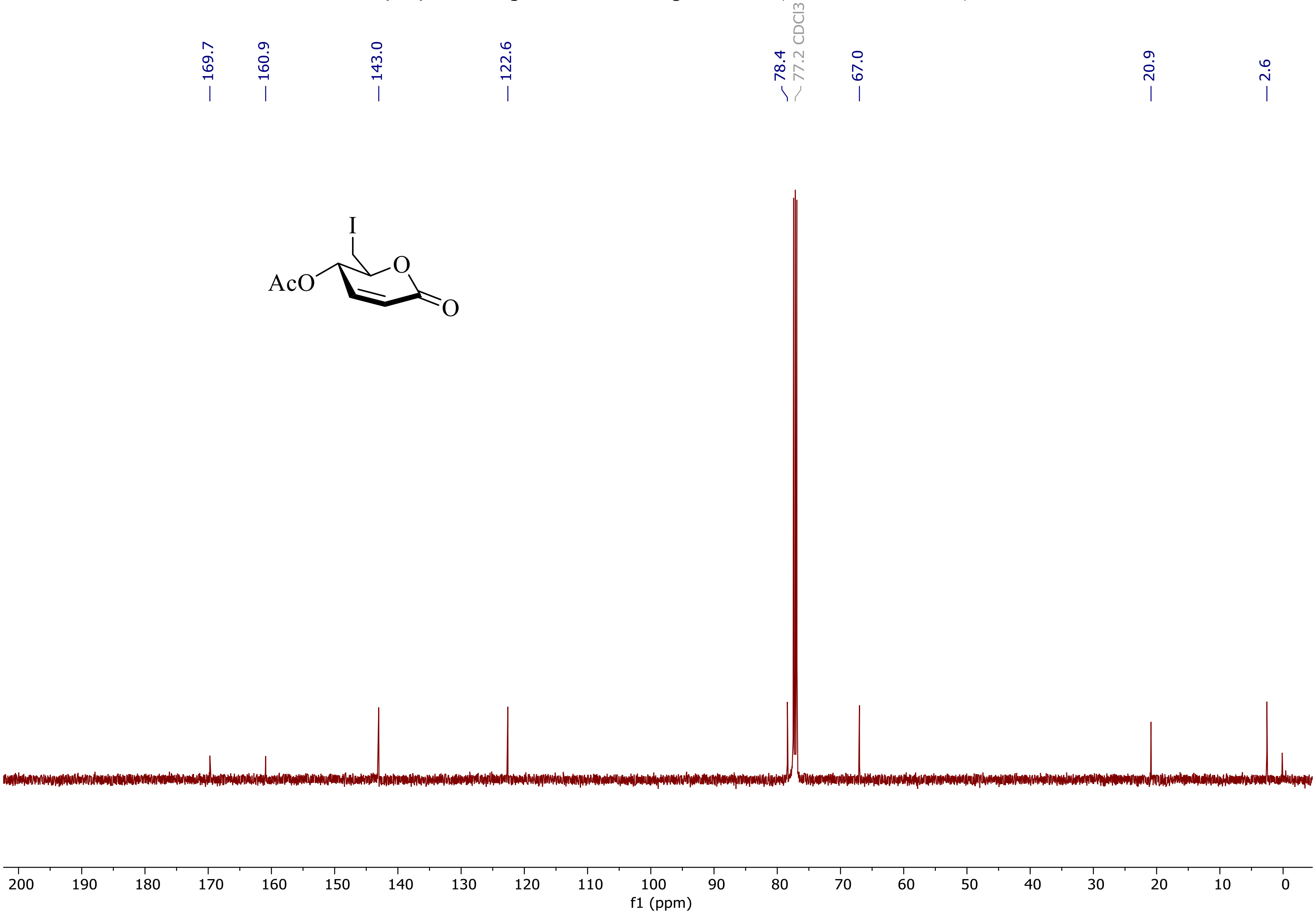

# $^{1}$H NMR spectrum of compound **17** (500 MHz, $CDCl_3$)

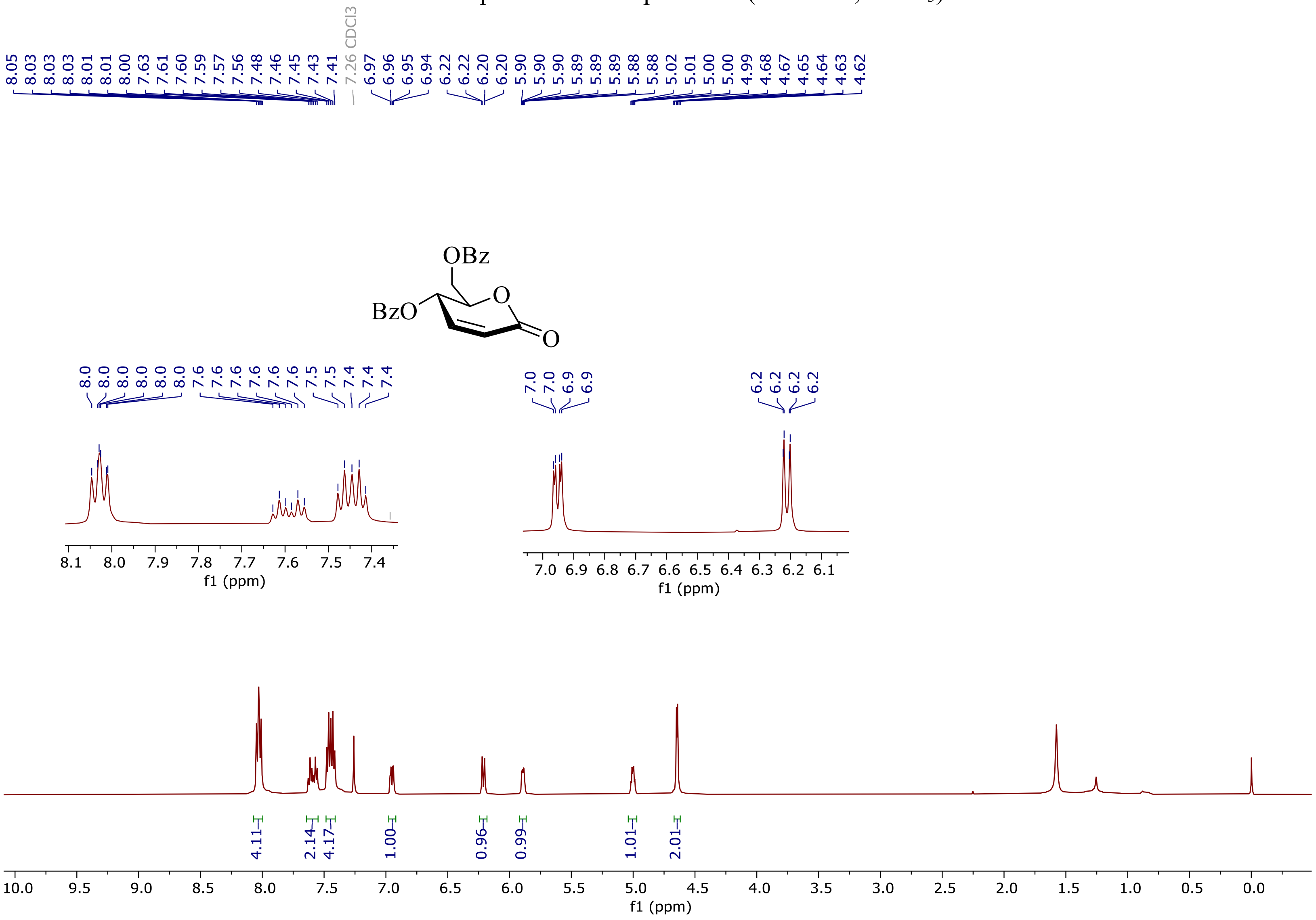

$^{13}C\{^1H\}$ NMR spectrum of compound **17** (125 MHz, $CDCl_3$)

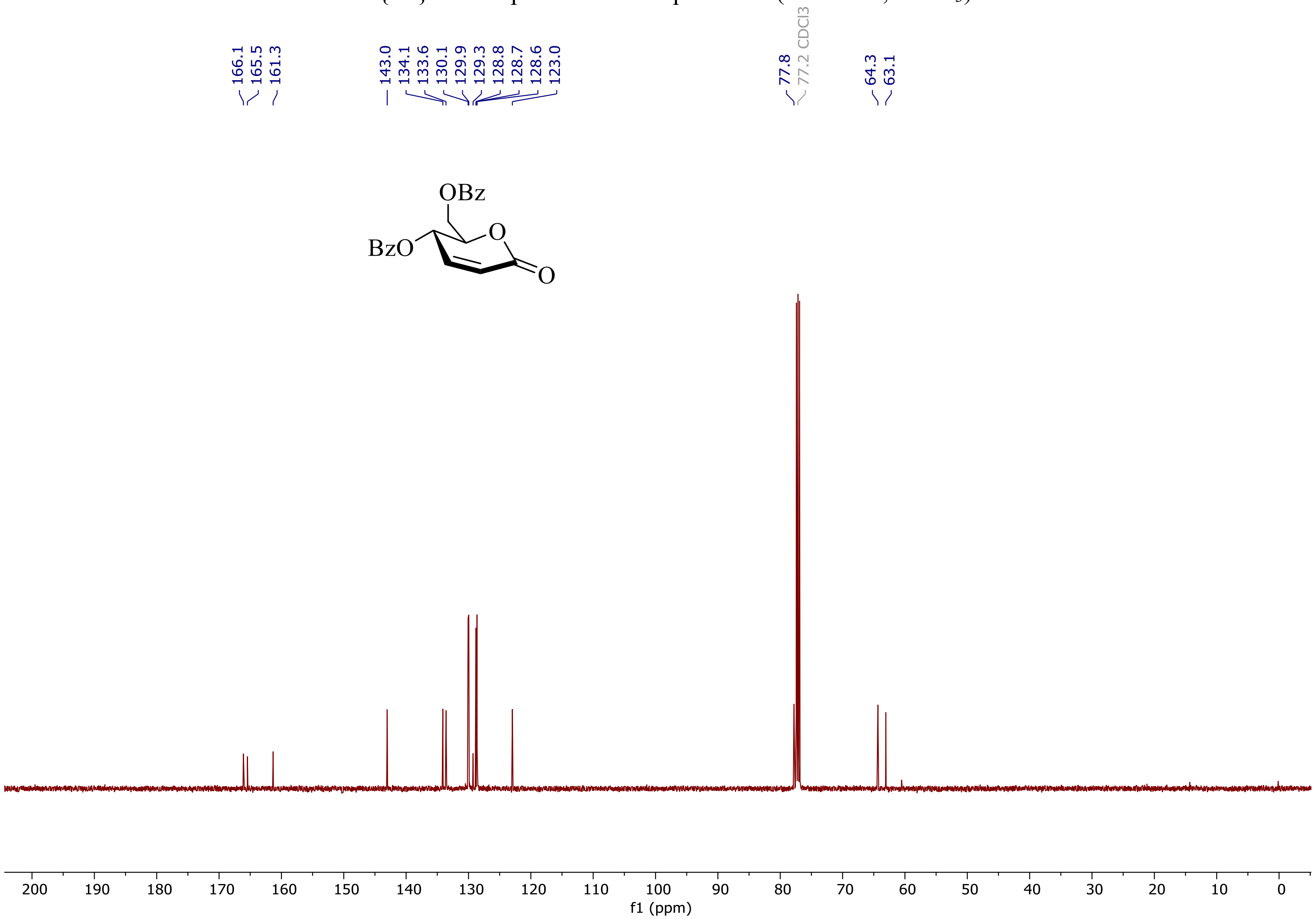

$^{1}$H NMR spectrum of compound **19** (500 MHz, $CDCl_3$)

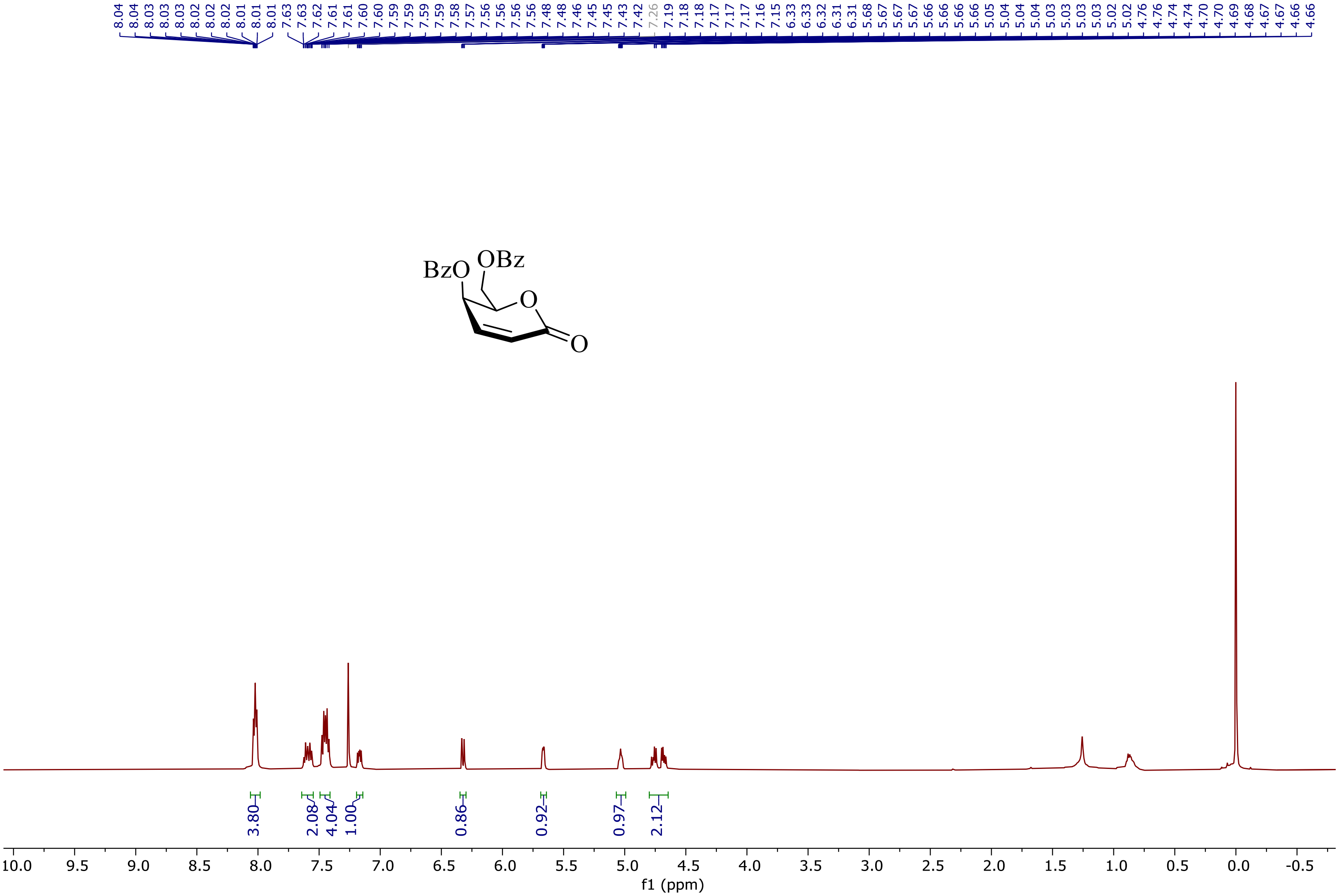

$^{13}C\{^{1}H\}$ NMR spectrum of compound **19** (125 MHz, $CDCl_3$)

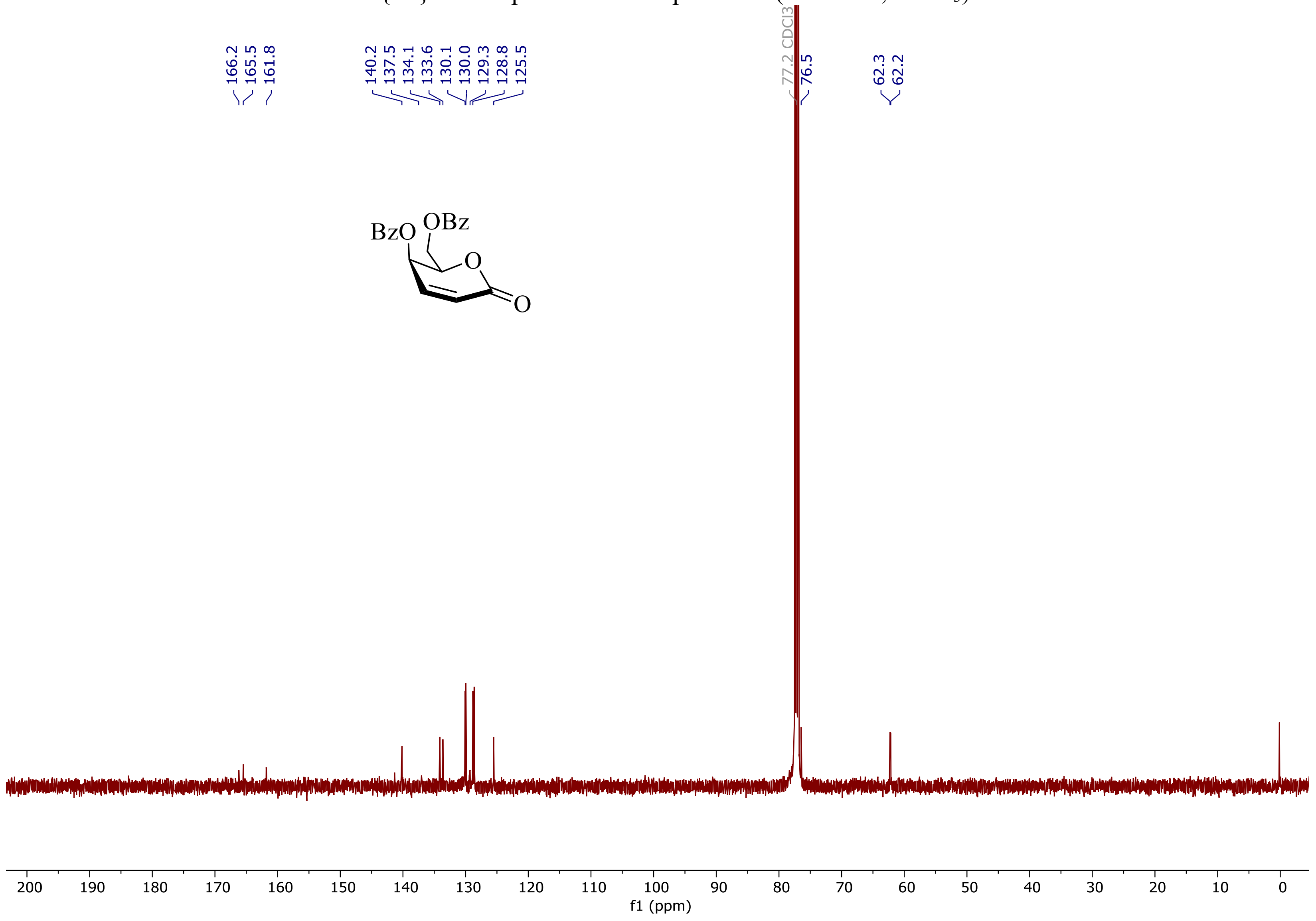

$^{1}$H NMR spectrum of compound **21** (500 MHz, $CDCl_3$)

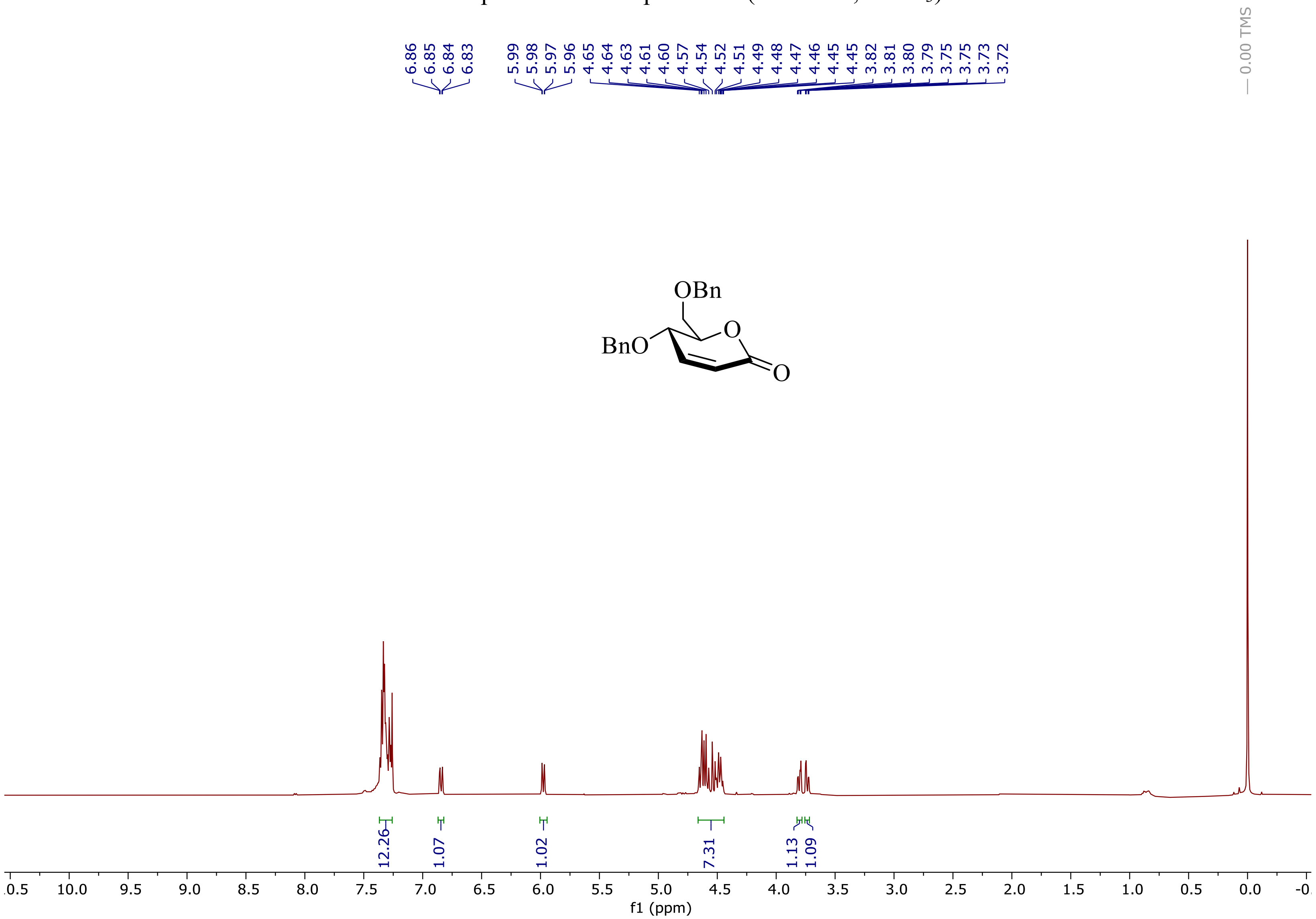

$^{13}C\{^1H\}$ NMR spectrum of compound **21** (125 MHz, $CDCl_3$)

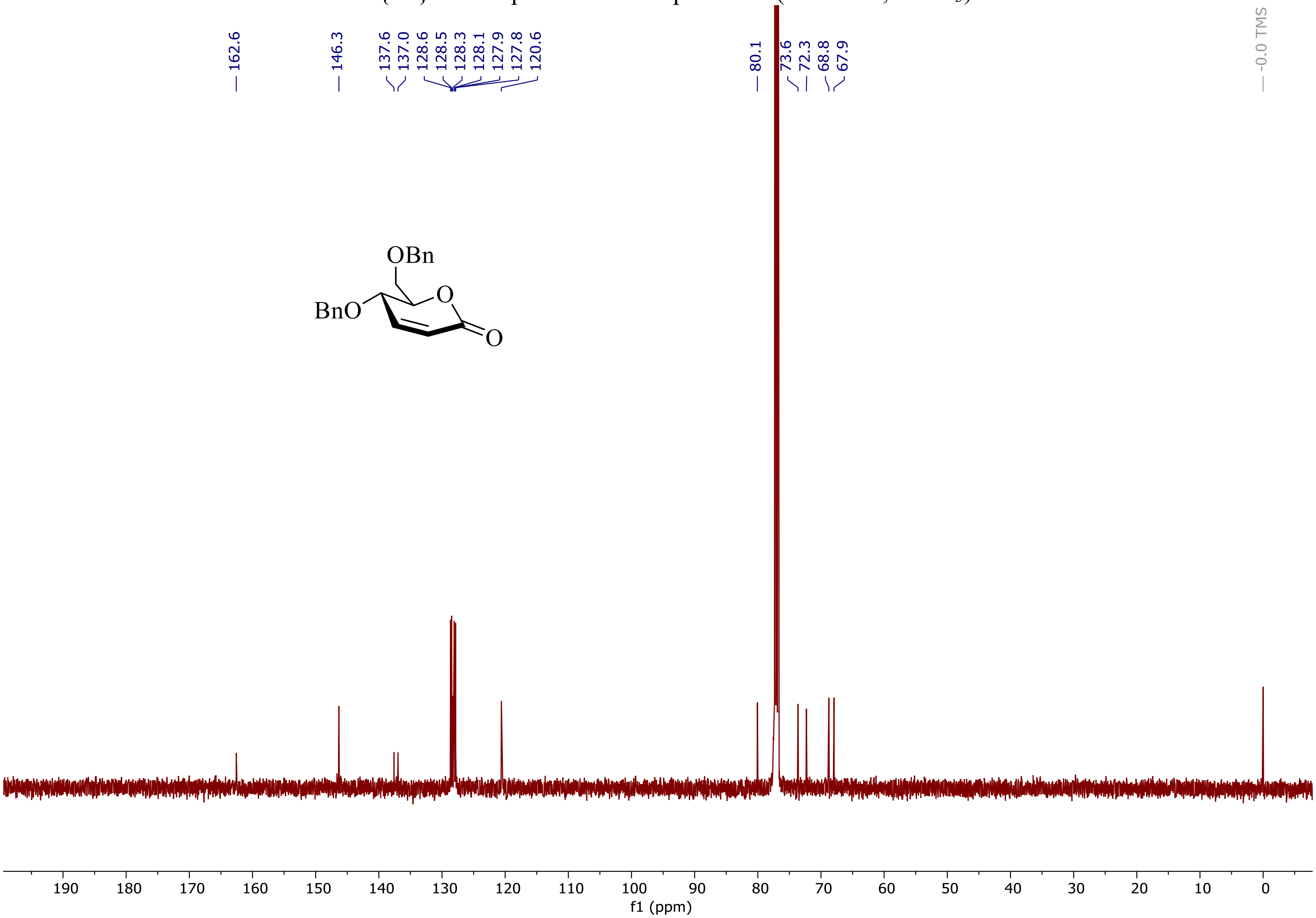

$^{1}$H NMR spectrum of compound **28** (500 MHz, $CDCl_3$)

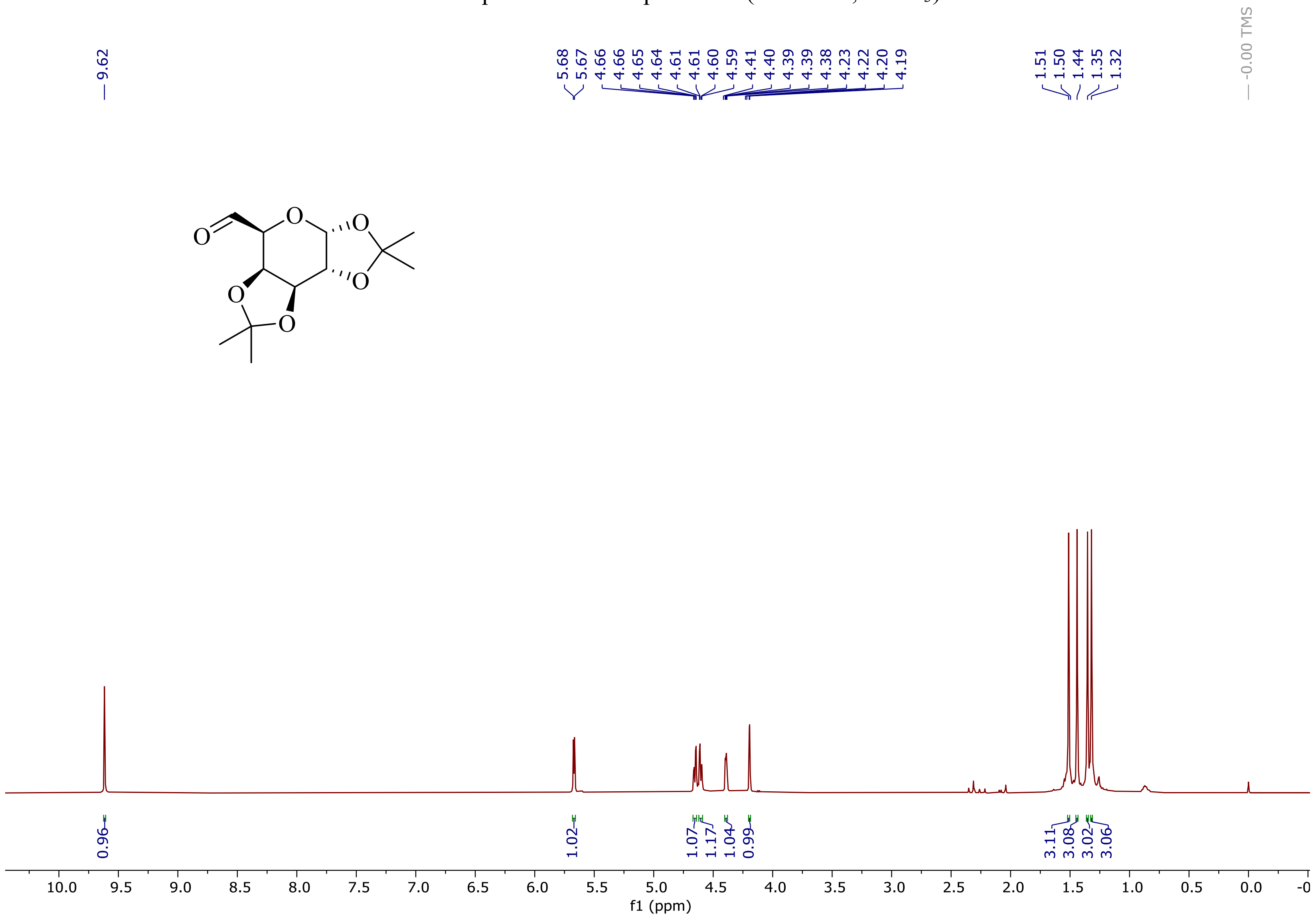

$^{13}C\{^{1}H\}$ NMR spectrum of compound **28** (125 MHz, $CDCl_3$)

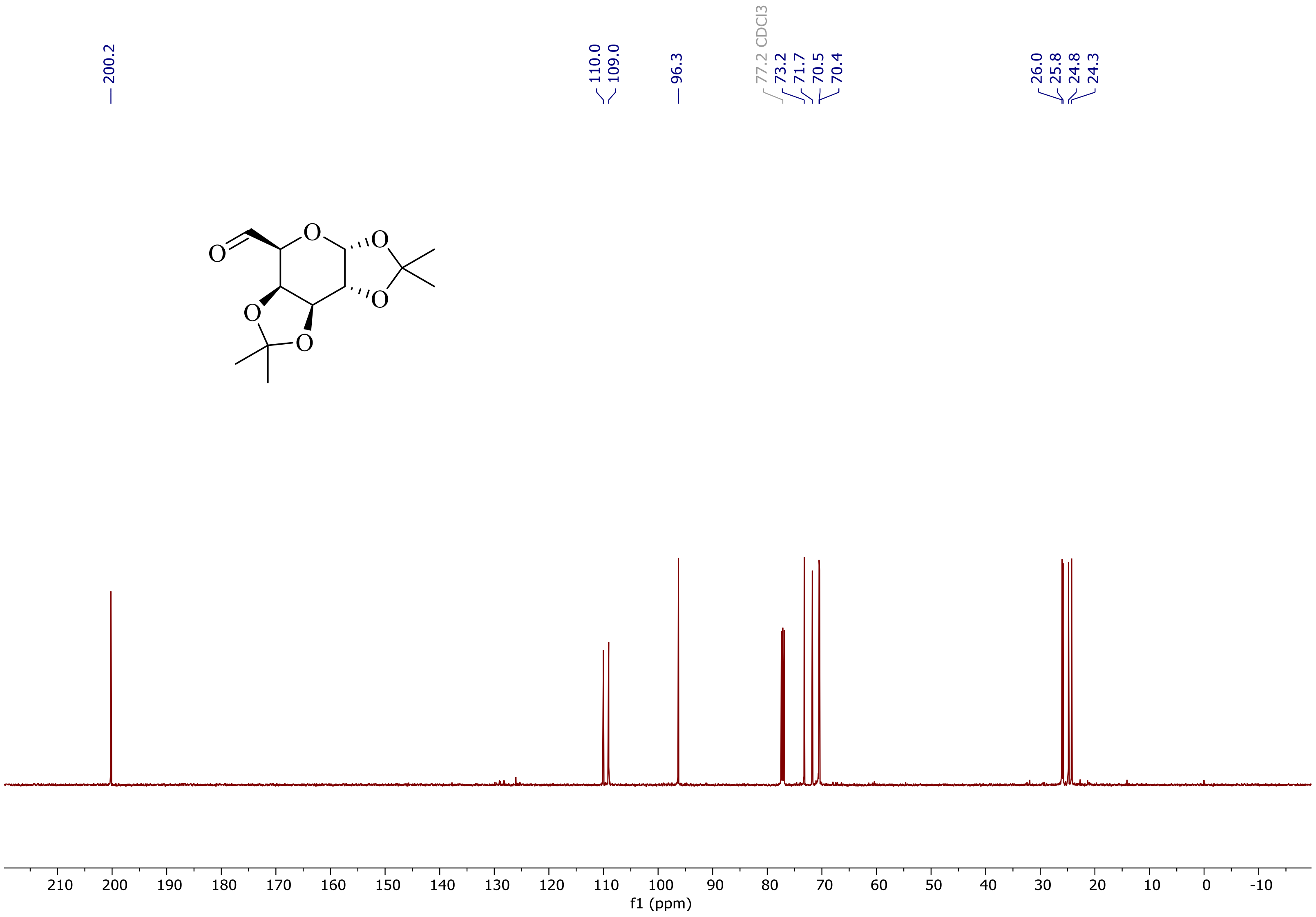

$^1$H NMR spectrum of compound **29** (500 MHz, $CDCl_3$)

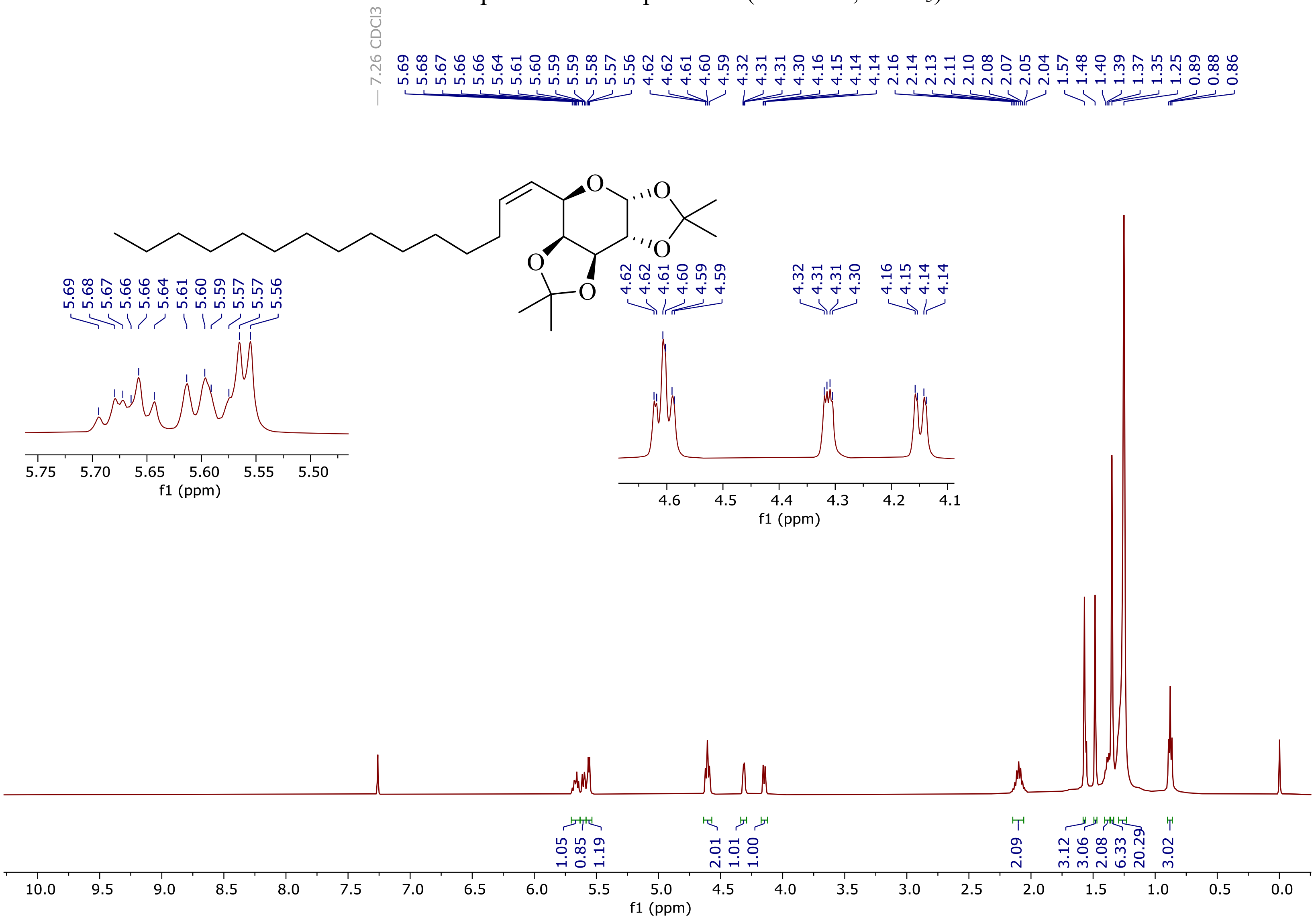

$^{13}C\{^{1}H\}$ NMR spectrum of compound **29** (125 MHz, $CDCl_3$)

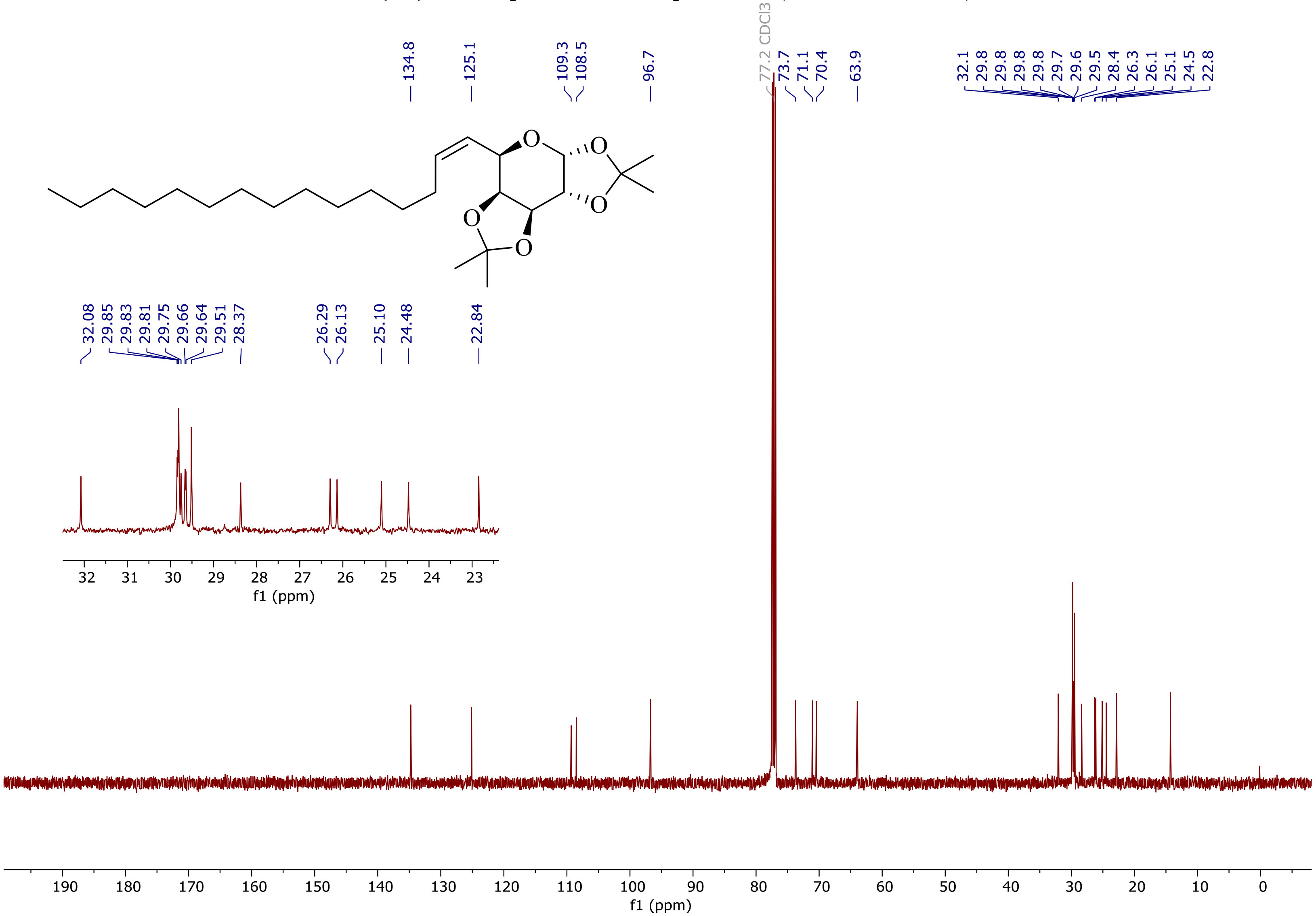

$^{1}$H NMR spectrum of compound **30** (500 MHz, $CDCl_3$)

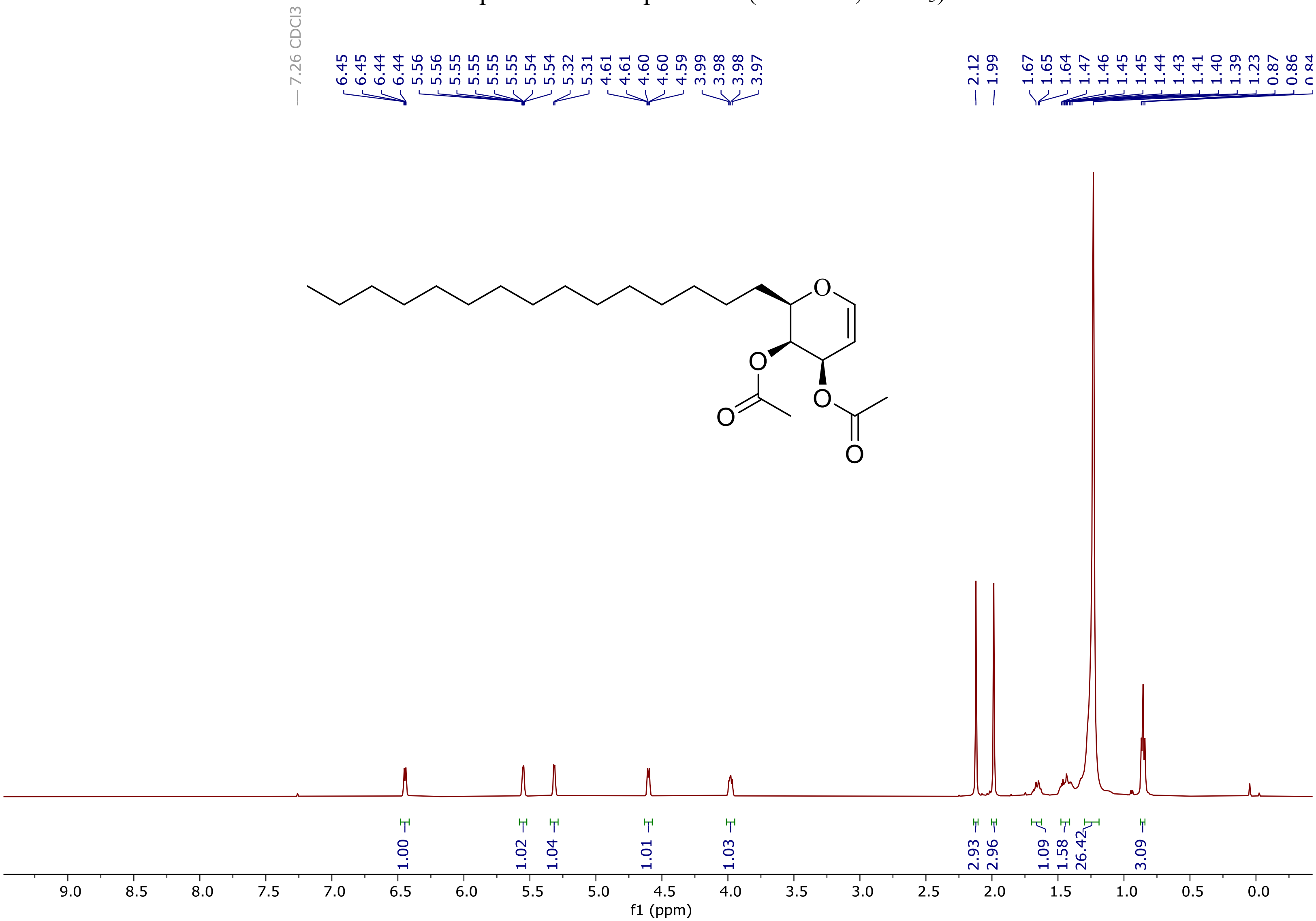

$^{13}C\{^1H\}$ NMR spectrum of compound **30** (125 MHz, $CDCl_3$)

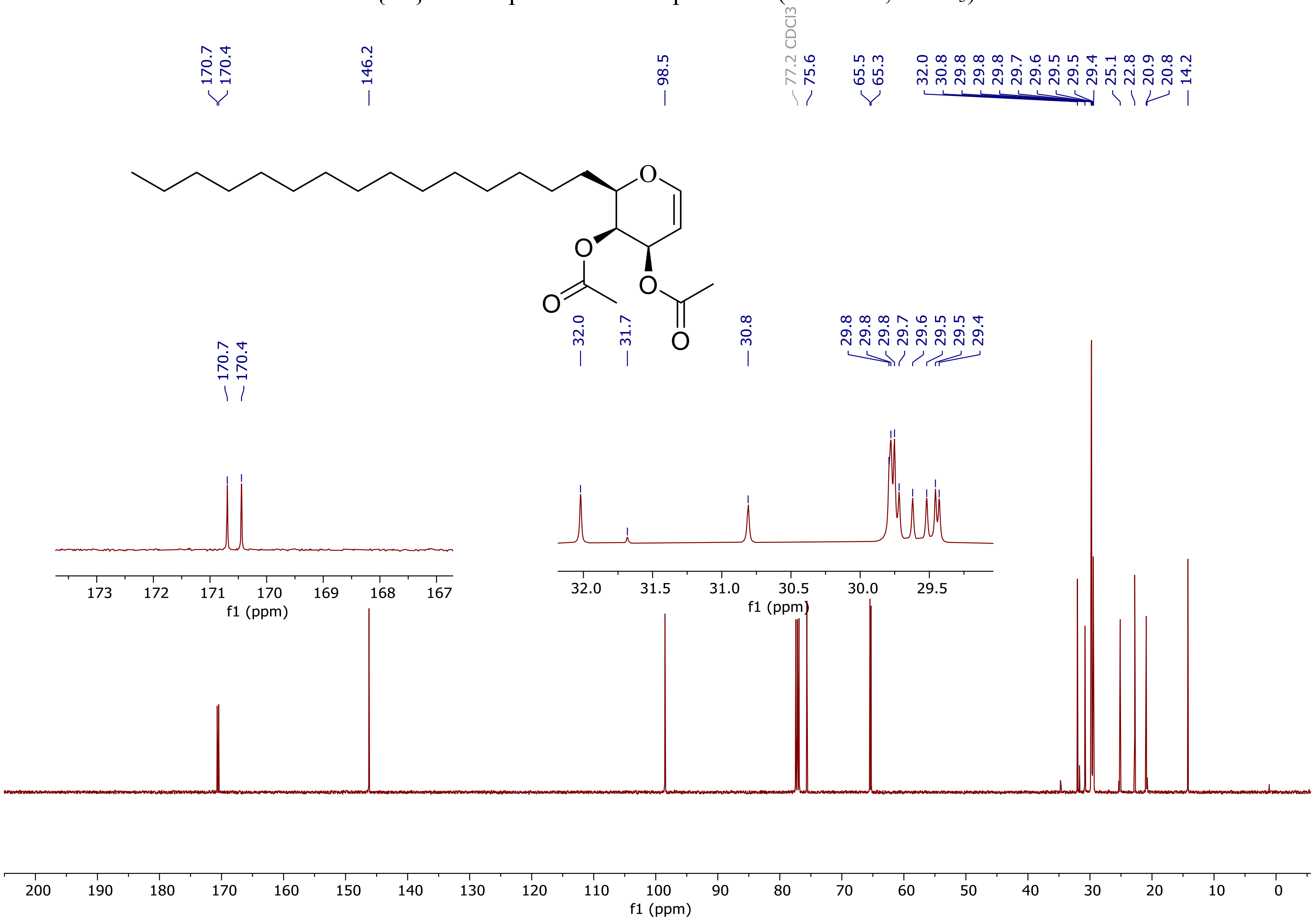

$^{1}$H NMR spectrum of compound **31** (500 MHz, $CDCl_3$)

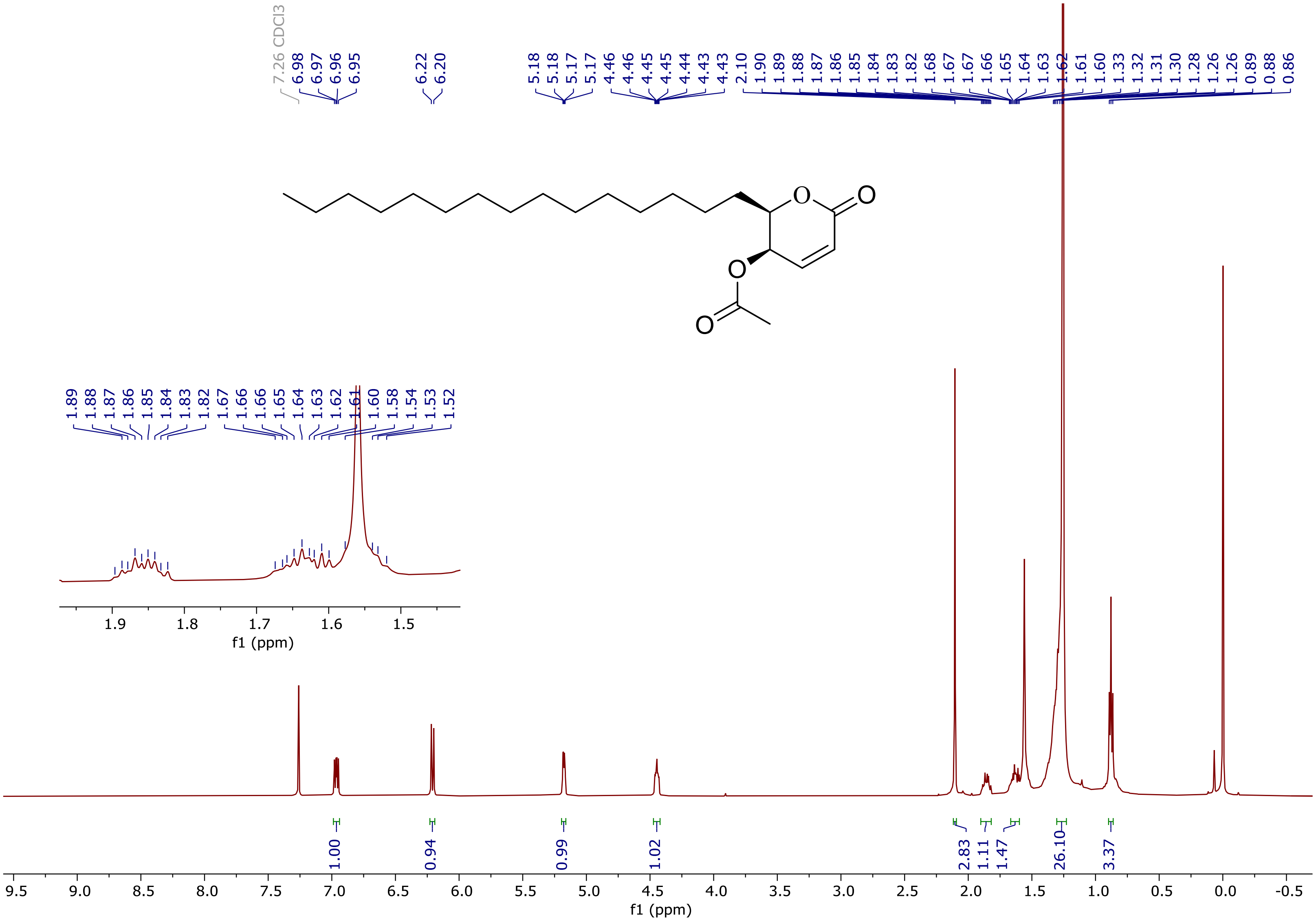

$^{13}C\{^1H\}$ NMR spectrum of compound **31** (125 MHz, $CDCl_3$)

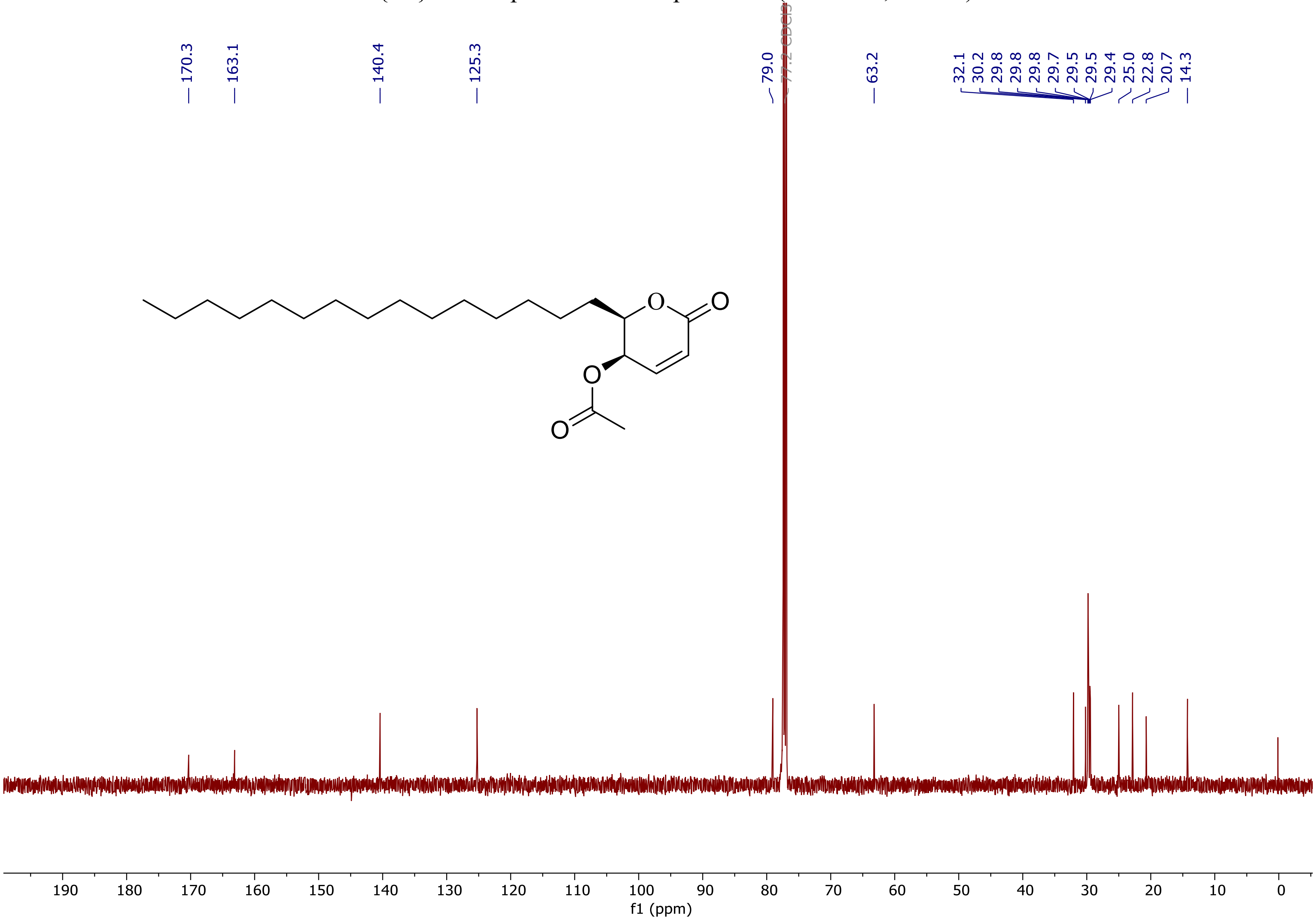

$^{1}$H NMR spectrum of **(-)-passifetilactone B** (500 MHz, $CDCl_3$)

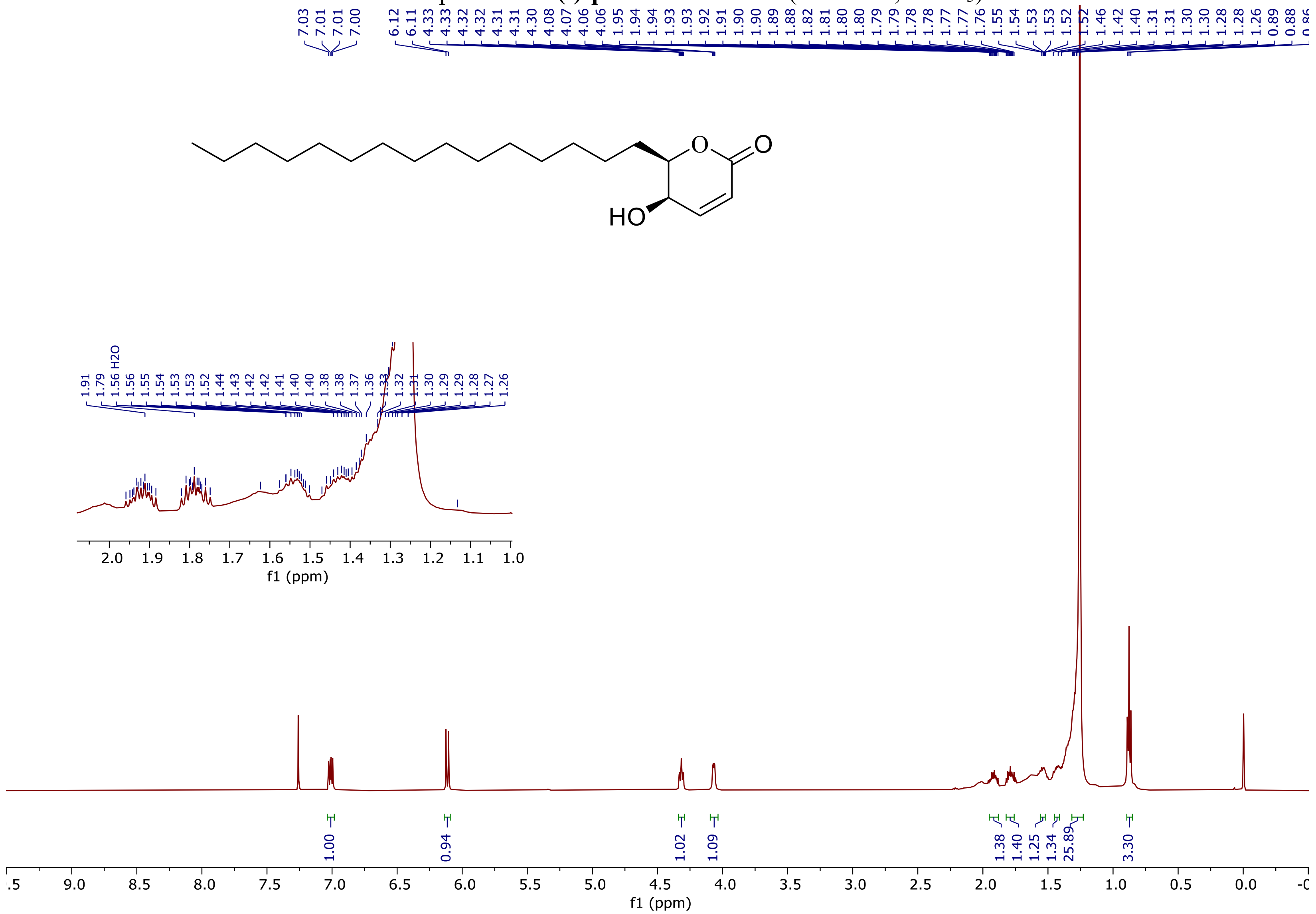

$^{13}C\{^{1}H\}$ NMR spectrum of **(-)-passifetilactone B** (125 MHz, $CDCl_3$)

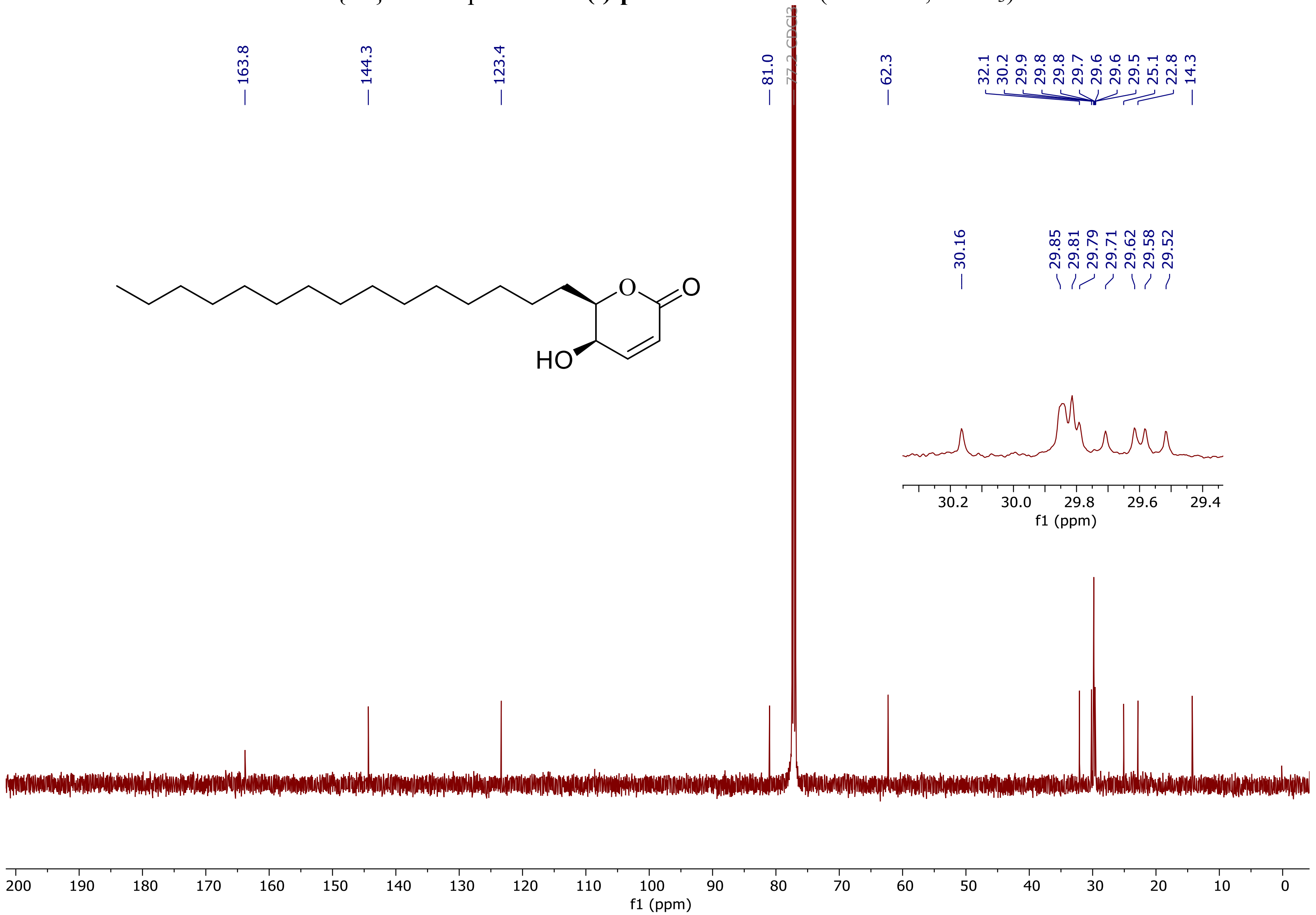

$^{1}$H NMR spectrum of **(-)-passifetilactone C** (500 MHz, $CDCl_3$)

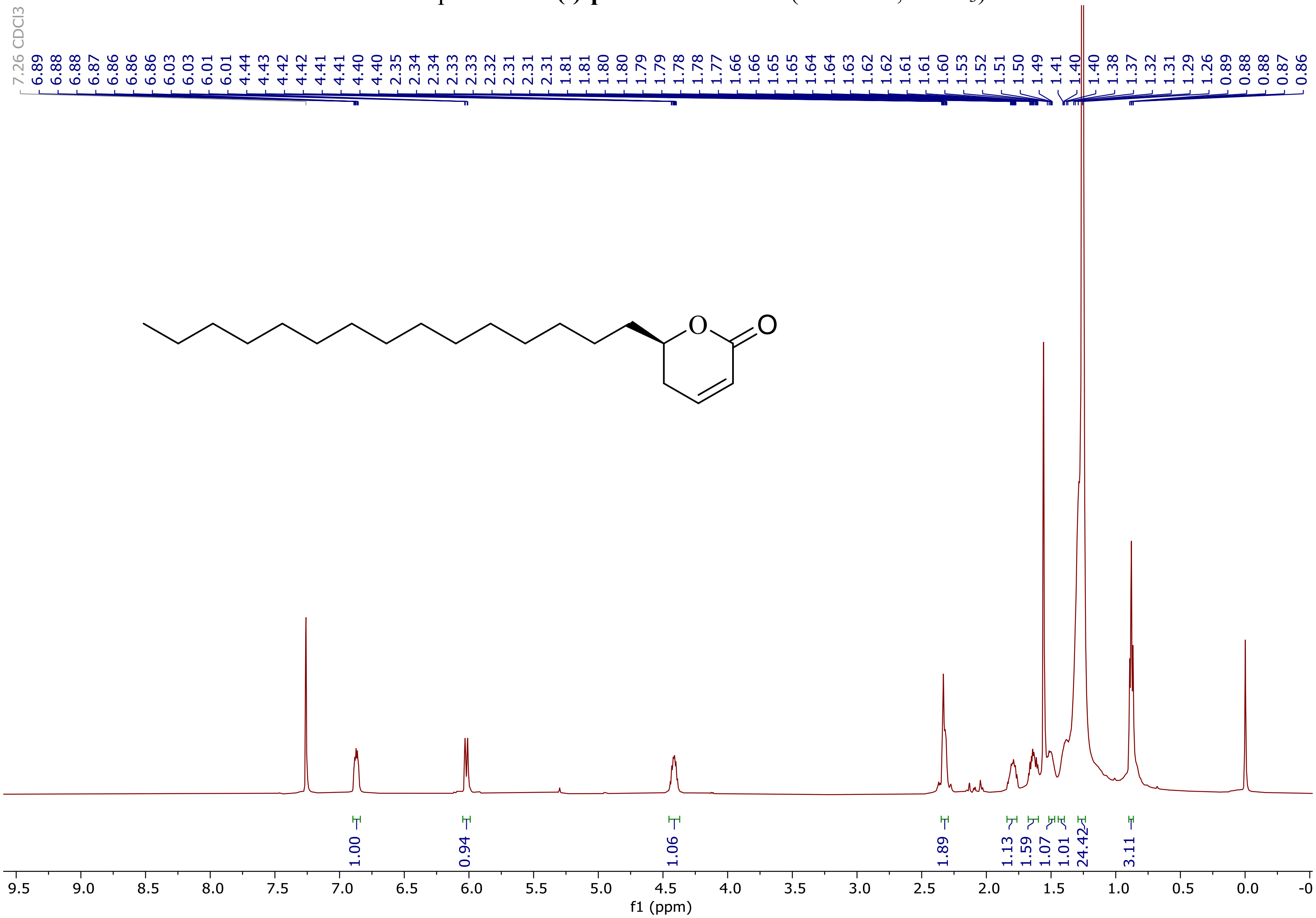

$^{13}C\{^{1}H\}$ NMR spectrum of **(-)-passifetilactone C** (125 MHz, $CDCl_3$)

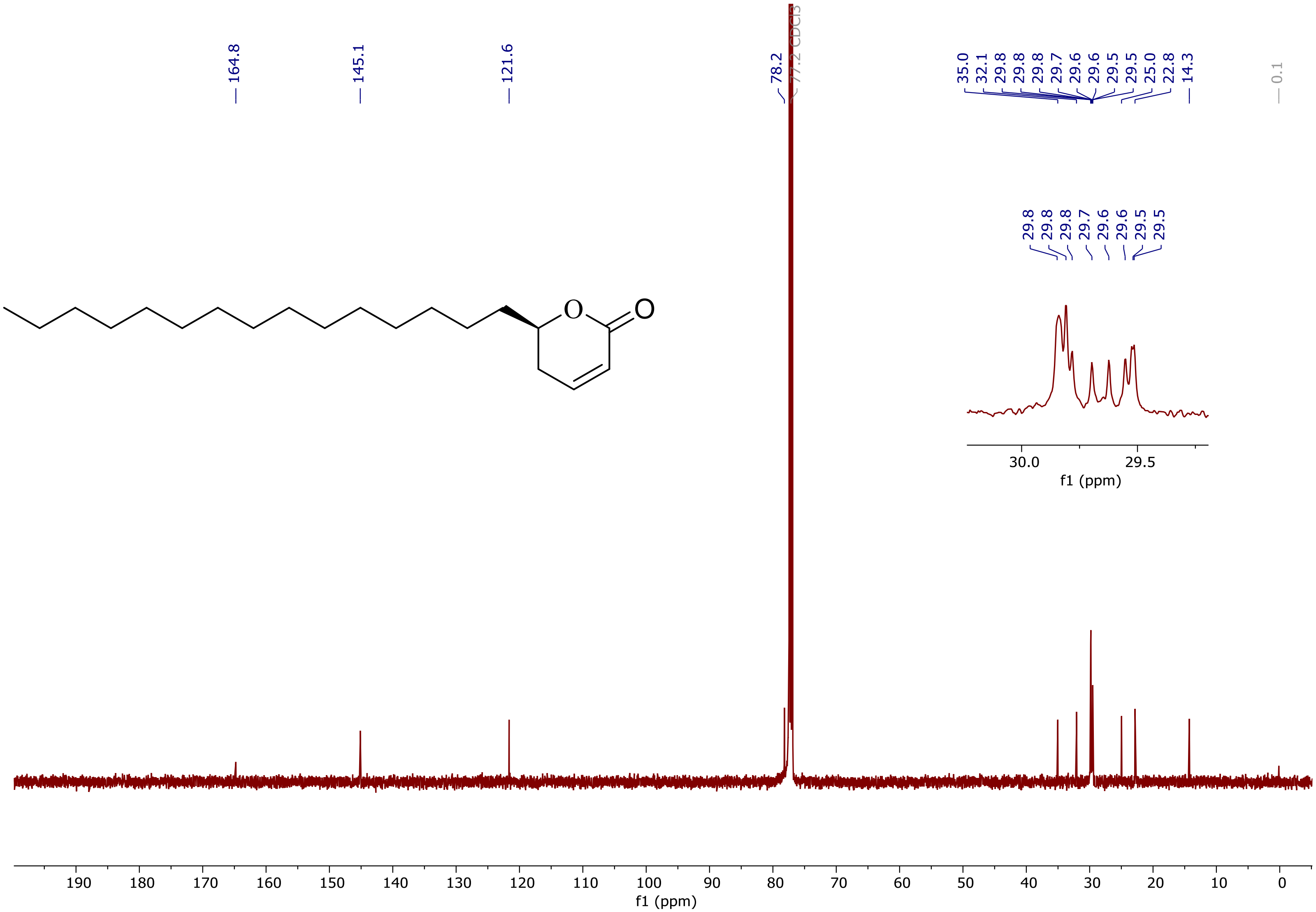

Supplement: Supplementary file 1 — jo5c00354_si_001.pdf [file jo5c00354_si_001.pdf]
